# Phylogenetic assessment of endangered and look‐alike Pigtoe species in a freshwater mussel diversity hotspot

**DOI:** 10.1002/ece3.9717

**Published:** 2023-01-24

**Authors:** Miluska Olivera‐Hyde, Jess W. Jones, Eric M. Hallerman

**Affiliations:** ^1^ Department of Fish and Wildlife Conservation Virginia Polytecnic Institute and State University Blacksburg Virginia USA; ^2^ U.S. Fish and Wildlife Service Blacksburg Virginia USA; ^3^ Present address: U.S. Geological Survey Eastern Ecological Science Center at the Leetown Research Laboratory Kearneysville West Virginia USA

**Keywords:** cryptic biodiversity, *Fusconaia*, Green River, Kentucky, mitochondrial DNA, *Pleurobema*, species boundaries

## Abstract

The Green River in Kentucky in the eastern United States is a freshwater mussel biodiversity hotspot, with 71 known species. Among them, the endangered *Pleurobema plenum* coexists with other morphologically similar species in the genera *Fusconaia* and *Pleurobema*, known colloquially as “pigtoes.” Identification of species in these genera is challenging even for mussel experts familiar with them. In our study, the correct identification of these species by experts ranged from 57% to 83%. We delineated taxonomic boundaries among seven species and tested for cryptic biodiversity among these look‐alike mussels utilizing mitochondrial and nuclear DNA sequence variation. Phylogenetic analysis of combined (1215 bp) mitochondrial DNA cytochrome oxidase I (*COI*) and NADH dehydrogenase 1 (*ND1*) genes showed five well‐diverged groups that included *F. flava*, *F. subrotunda*, *P. cordatum*, and *P. plenum* as distinct clades, with *P. sintoxia* and *P. rubrum* grouped into a single clade. While our mitochondrial DNA analyses did not distinguish *P. sintoxia* and *P. rubrum* as phylogenetically distinct species, the typical shell forms of these two nominal taxa are very distinct. Further phylogenetic analysis using nuclear ribosomal transcribed spacer region subunit I (*ITS1*) DNA sequences also showed that *P. sintoxia* and *P. rubrum* were not distinct lineages. No cryptic species were detected in the *Fusconaia* and *Pleurobema* samples analyzed from the Green River. The highest haplotype diversity (*h*), average number of nucleotide differences (*k*), and nucleotide diversity (*π*) were observed for *F. subrotunda* at both the *COI* (*h* = 0.896, *k* = 3.805, *π* = 0.00808) and *ND1* (*h* = 0.984, *k* = 6.595, *π =* 0.00886) markers, with similarly high genetic diversity in the other taxa. Our results give managers confidence that cryptic taxa do not occur within or among these morphologically similar species in the Green River, and populations appear genetically diverse, indicative of large and healthy populations.

## Graphical abstract

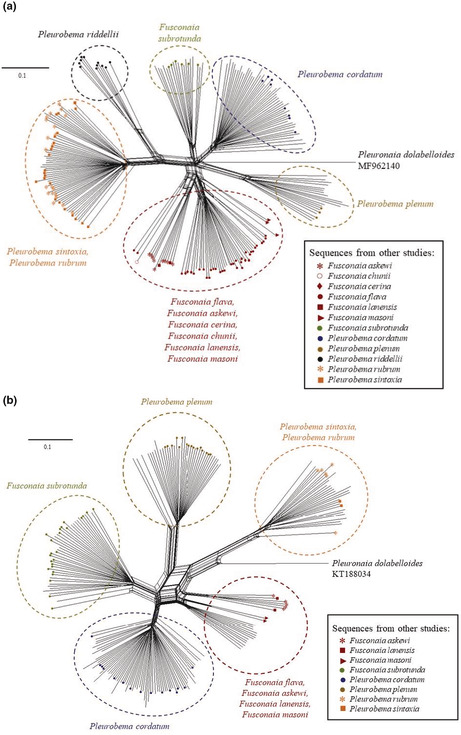

## 1 INTRODUCTION

Freshwater mussels of the Family Unionidae comprise 679 species, with a global hotspot of diversity in North America and other areas of high diversity in South America and southeast Asia (Lopes‐Lima et al., 2018). In particular, the southeastern United States is an area of high species richness, endemism, and imperilment (Elkins et al., 2019). Understanding of the systematics of unionids mussels is still in flux (Graf & Cummings, 2007), and cryptic, species‐level variation is still becoming recognized; for example, Schilling (2015) found evidence of several cryptic species in the genus *Pleurobema* and *Pleuronia* in the upper Tennessee River basin. Hence, it is important to investigate and recognize species limits in order to inform conservation actions. The so‐called pigtoe mussels (Tribe Pleurobemini; genera *Fusconaia*, *Pleurobema*, and *Pleuronaia*), which are broadly distributed in river systems in central and eastern North America (Campbell & Lydeard, 2012b; Heard & Guckert, 1970), are such a group of freshwater mussels (Campbell & Lydeard, 2012a, 2012b; Inoue et al., 2018; Morrison et al., 2021; Schilling, 2015), and those of the Ohio River basin are yet to receive sufficient phylogenetic characterization.

**FIGURE 1:**
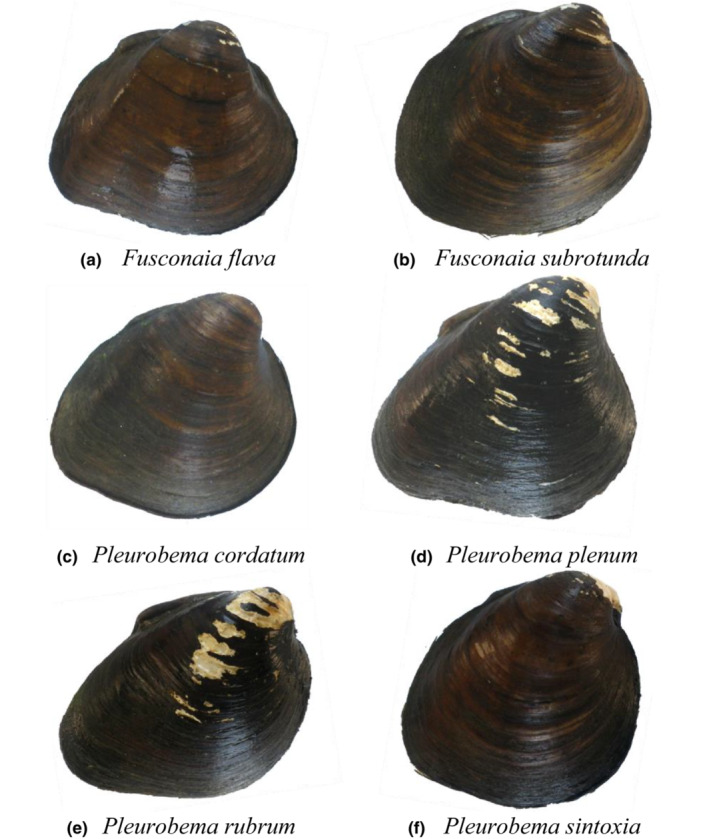
Representative shell forms for: (a) *Fusconaia flava*, (b) *Fusconaia subrotunda*, (c) *Pleurobema cordatum*, (d) *Pleurobema plenum*, (e) *Pleurobema rubrum*, and (f) *Pleurobema sintoxia*. These mussel specimens were identified consistently as these shell forms by all experts. Mussel specimens were collected in 2015 from Pool 4 (GPS coordinates = 37.18286, −86.6296; river mile = 149) in the Green River, Kentucky.

**FIGURE 2:**
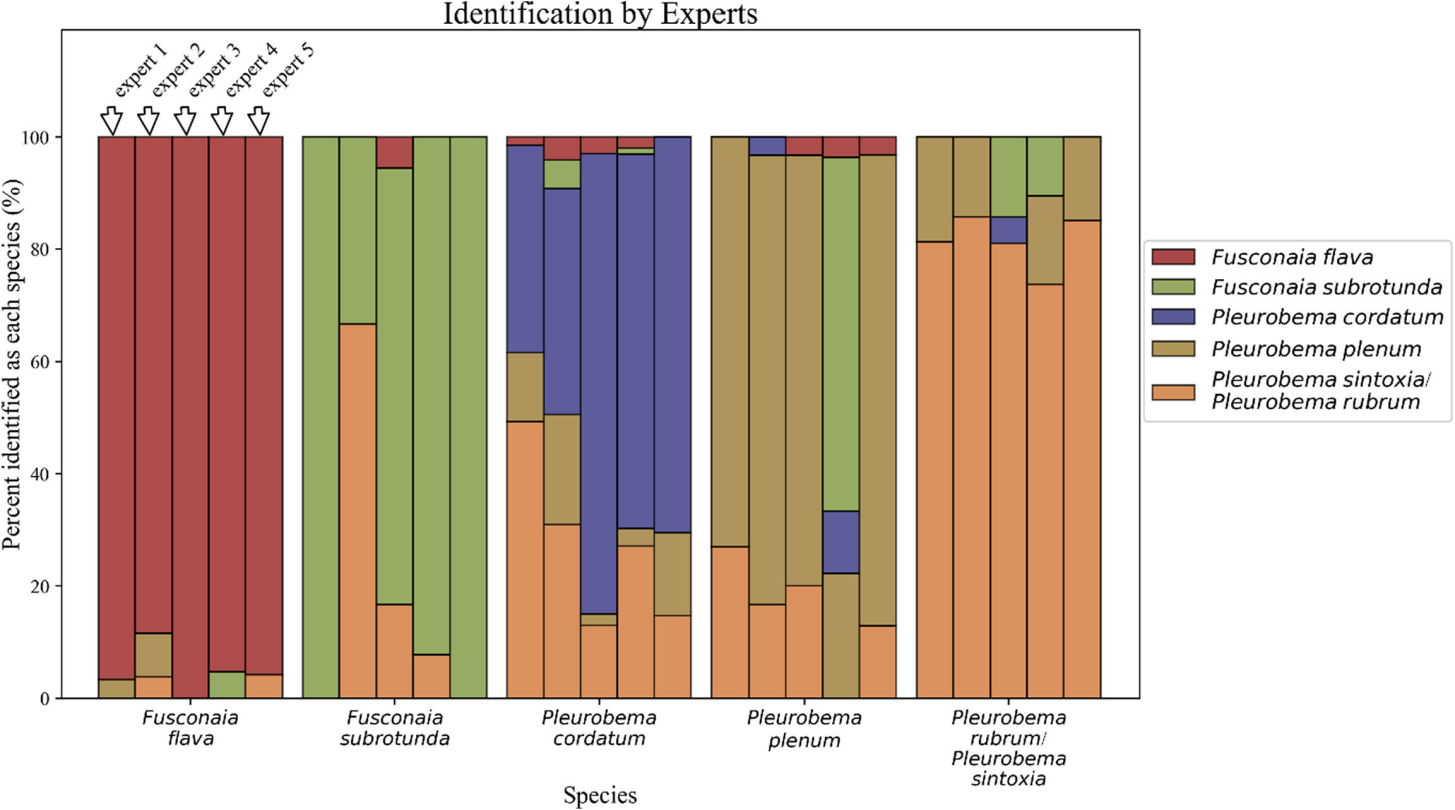
Bar graph showing percentages of correctly identified and misidentified individuals for each species of *Fusconaia* and *Pleurobema* by five experts. On average, the experts were able to correctly identify the mussels 70% of the time. Mussel specimens were collected in 2015 from Pool 4 (GPS coordinates = 37.18286, −86.6296; river mile = 149) in the Green River, Kentucky.

The Green River, Kentucky (KY) has one of the most diverse assemblages of mussels in the United States, including seven species of *Fusconaia* and *Pleurobema*. All seven pigtoe species are of conservation management concern (Master et al., 1998), with IUCN Red List status as: critically endangered for rough pigtoe (*P. plenum*; Bogan, 1996a) and Clubshell (*P. clava*; Bogan, 1996b), vulnerable for long‐solid (*F. subrotunda*; Cummings & Cordeiro, 2012a), near‐threatened for Ohio pigtoe (*P. cordatum*; Bogan & Seddon, 1996) and pink pigtoe (*P. rubrum*; Bogan, 1996c), and least‐concern for Wabash pigtoe (*F. flava*; Cummings & Cordeiro, 2011) and round pigtoe (*P. sintoxia*; Cummings & Cordeiro, 2012b). Complicating their management, the shell phenotypes of these species are very similar (Figure 1). Notably, five mussel experts, familiar specifically with pigtoes, identified several pigtoes individuals collected from the Green River. Individuals of *F. flava* were the easiest to identify by the experts (Figure 2). *Fusconaia subrotunda* had a high rate of misidentification and species in the genus *Pleurobema* were the most challenging to identify and frequently confused among each other. Such results show that pigtoes in the Green River are particularly difficult to distinguish morphologically even by experts. Previous studies describing these species focused on a suite of shell and soft‐body morphological characters. For example, some of the principal differences among species of *Fusconaia* and *Pleurobema* are the number of gills charged when gravid (four for *Fusconaia* and two for *Pleurobema*), conglutinate morphology (leaflike for *Pleurobema* and subcylindrical for *Fusconaia*), and foot color (white for *Pleurobema* and generally orange for *Fusconaia*). However, these shell and soft‐body characters can overlap, depending on environmental and genetic factors, mussel age and size, and phenotypic plasticity (Inoue et al., 2014), and are difficult to characterize on living individuals or are not expressed in all seasons. In addition, similar phenotypes could be the result of all these Pleurobemini species being closely related phylogenetically, resulting in these species sharing characters.

**FIGURE 3:**
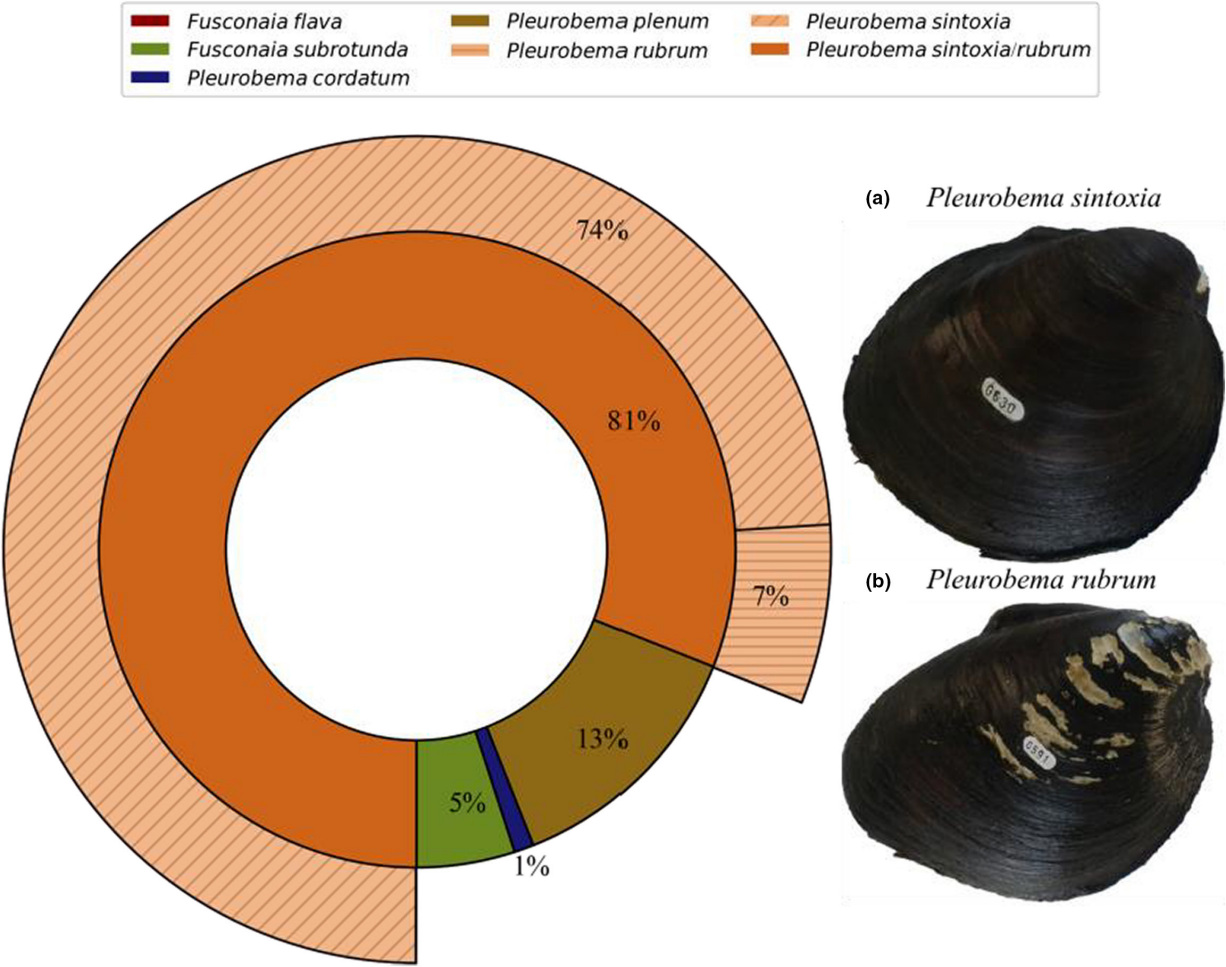
Pie chart showing experts' field identification for mussel specimens that where morphologically identified to the clade *Pleurobema sintoxia*/*rubrum*. Two representative shells for (a) *Pleurobema rubrum* and (b) *Pleurobema sintoxia* shell forms. These mussel specimens were identified consistently as these two shell forms by all the experts. Mussel specimens were collected in 2015 from Pool 4 (GPS coordinates = 37.18286, −86.6296; river mile = 149) in the Green River, Kentucky.

Recent advances in development of molecular markers and the widening application of phylogenetic analyses have led to more reliable identification of freshwater mussels and to development of more scientifically defensible management plans. Phylogenetic analysis of freshwater mussels in North America has focused increasingly on application of DNA sequence variation, including variation at the mitochondrial DNA (mtDNA) *16S rRNA*, cytochrome oxidase subunit I (*COI*), cytochrome *b* (*Cyt*‐*b*), and NADH dehydrogenase 1 (*ND1*) genes, as well as at nuclear genes, including large ribosomal subunit *28S rDNA* and the ribosomal internal transcribed spacer region subunit 1 (*ITS1*) (Campbell et al., 2008; Campbell & Lydeard, 2012a; Graf & Cummings, 2007; Jones et al., 2015). Several recent studies have characterized phylogenetic relationships among species in the genera *Fusconaia* and *Pleurobema* using these markers. For example, *F. flava*, *F. cerina*, and *F. askewi* were shown to be the same phylogenetic species based on *COI* and *ND1* haplotypes (Campbell & Lydeard, 2012a). Further, species in the genus *Fusconaia* tend to show low intrapopulation (*F. subrotunda*) and low interpopulation (*F. flava*/*cerina*) variation, i.e., mtDNA divergence is low both within and among species in this genus (Campbell & Lydeard, 2012a). For mussel species in the Green River, phylogenetic relationships among *F. flava*, *F. subrotunda*, *P. cordatum*, *P. clava*, *P. plenum*, *P. sintoxia*, and *P*. *rubrum* were recently assessed using *CO1*, *ND1*, and *ITS1* markers (Campbell et al., 2008; Campbell & Lydeard, 2012b; Inoue, 2018; Jones et al., 2015). Early studies suggested the existence of a *P. cordatum* group which included *P. cordatum*, *P. plenum*, *P. rubrum*, and *P. sintoxia* (Campbell et al., 2008; Campbell & Lydeard, 2012b), and more extensive sampling by Inoue et al. (2018) and Jones et al. (2015) validated the interpretation that *P. cordatum* and *P. plenum* are indeed different species. Utilizing *ND1* and *COI* markers, these authors showed that only a few nucleotide differences exist between individuals of *P. sintoxia* and *P. rubrum* and found that further phylogenetic assessment was needed to delineate these two nominal taxa. While the shells of *P. sintoxia* generally are morphologically distinctive, they can occasionally be mistaken for *P. rubrum* and vice‐versa (Figure 3). However, the umbos of *P. rubrum* are pointed and pronounced, resembling a pyramid, hence the common name “Pyramid pigtoe.” Further, this species typically has a well‐defined sulcus traversing the middle of each valve, especially in larger and older mussel specimens (Miller et al., 2008). Another relevant example of closely related species is *P. clava* and *P. oviforme*, species endemic to the Tennessee and Cumberland River watersheds, for which Campbell et al. (2008) showed few molecular differences at mtDNA genes between these taxa, although when assessed at nuclear *ITS1*, differences were observed. More recently, Morrison et al. (2021) conducted a rang‐wide assessment of mitochondrial (mtDNA) and nuclear microsatellite DNA and showed minimal mtDNA genetic divergence and even some haplotype sharing over wide geographic areas between the two taxa throughout the Ohio River basin, but very high divergence at microsatellite markers and distinctive morphology for a population occurring in the extreme headwaters of the upper Tennessee River basin. Therefore, utilization of nuclear as well as mitochondrial DNA sequences is critical for assessing phylogenetic differentiation among closely related species.

Phylogenetic relationships among species in the genera *Fusconaia* and *Pleurobema* have been assessed across various geographic regions in North America using a suite of molecular markers. While these comparisons have been made with a large number of specimens from various species belonging to the Tribe Pleurobemini (Campbell et al., 2008; Campbell & Lydeard, 2012a, 2012b; Graf & Cummings, 2007; Jones et al., 2015), molecular data are sparse for these species in the Green River, Kentucky. Rigorous phylogenetic assessment of morphologically similar species in these two genera would be vital to support development of a probabilistic dichotomous key for the Green River and the regional Ohio River mussel faunas. Further, an in‐depth phylogenetic identification of mussels in the genera *Fusconaia* and *Pleurobema* from the Green River utilizing large sample sizes, would help determine whether any cryptic species occur in the river, and assist with the design and implementation of appropriate management plans for imperiled and critically endangered species, especially for *P. plenum*.

## 2 MATERIAL AND METHODS

### 2.1 Sample collection

**FIGURE 4:**
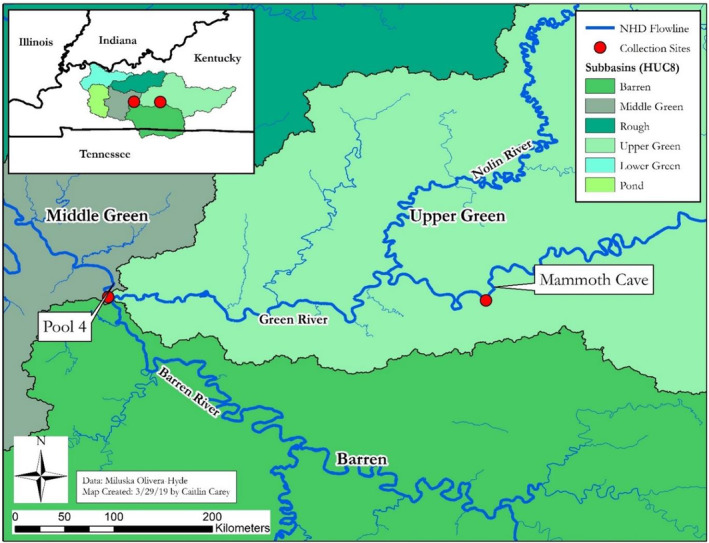
Sampling locations for freshwater mussel species in the genera *Fusconaia* and *Pleurobema*. Mussel specimens were collected in 2015 and 2017 from Pool 4 (GPS coordinates = 37.18286, −86.6296; river mile = 149) and mammoth cave National Park (GPS coordinates 37.17819, −86.1154; river mile = 197) in the Green River, Kentucky.

A total of 258 mussel specimens belonging to species in the genera *Fusconaia* and *Pleurobema* were collected from two sites in the Green River, KY, Pool 4 during September 2015 (GPS coordinates = 37.18286, −86.6296; river mile = 149; Figure 4). A second sampling effort was conducted to increase the number of mussel specimens in the respective size ‐classes, especially of smaller mussel specimens. This second sampling occurred in the Western Kentucky University BioPreserve just upstream of Mammoth Cave National Park (GPS coordinates 37.17819, −86.1154; river mile = 197) during November 2017. An additional 17 individuals were collected from the Clinch River at an unnamed site near Sneedville (GPS coordinates 36.523311, −83.204240), Frost Ford (GPS coordinates 36.534881, −83.179205), and Kyle's Ford (GPS coordinates 36.565230, −83.054863), TN. In addition, five individuals were collected at other locations in the Tennessee River downstream of Pickwick Dam, Hardin County, TN. These individuals were collected for use as outgroups to test for any cryptic species among collections from distinct watersheds. All mussel specimens collected from the Green River were physically tagged and kept at the Minor E. Clark Fish Hatchery, near Morehead, Kentucky, until data from the shell morphological analysis were collected. For each species, tissue for DNA isolation was collected nonlethally by swabbing the mussel foot with a DDK‐50 swab (Isohelix, Harriettsham, UK). Five mussel experts identified specimens of *Fusconia* and *Pleurobema* collected from the Pool 4. These individuals had been identified to species using molecular characters established in this study.

### 2.2 Polymerase chain reaction

We extracted DNA using an Isohelix (Harrietsham) DNA Isolation Kit. Concentration and purity of the double‐stranded DNA were measured using a μLite PC spectrophotometer (Biodrop), and DNA was diluted to 10–30 ng/μl. All polymerase chair reactions (PCRs) were performed in either a T100™ or MyCycler™ thermocycler (both from Bio‐Rad). PCR products were sent to the Fralin Life Sciences Institute (Blacksburg, VA) for Sanger sequencing. For *ND1*, we used two different pairs of primers to obtain amplified sequences for all species (Table 1). Amplification products were obtained for most mussel specimens of *F. flava*, *F. subrotunda*, *P. cordatum*, and *P. plenum* using primers *Leu‐uurF* and *LoGlyR* (Serb et al., 2003). For some mussel specimens (later identified as *P. sintoxia and P. rubrum*), it was necessary to use primers *nadh1‐F* and *nadh1‐R* (Buhay et al., 2002; Serb & Lydeard, 2003). Details regarding PCR amplification appear in Supplmental Material S1.

**TABLE 1:** Mitochondrial DNA primers for NADH dehydrogenase 1 (*ND1*) and cytochrome oxidase subunit I (*COI*), nuclear DNA primers for the ribosomal internal transcribed spacer region subunit 1 (*ITS1*) used for amplification of DNA sequences and genetic analysis of freshwater mussel species belonging to the genera *Fusconaia* and *Pleurobema* in the Green River, Kentucky.

Gene	Primer name	Sequence	Reference
*ND1*	*Le‐uuurF*	F: 5′‐TGG CAG AAA AGT GCA TCA GAT TAA AGC‐3′	Serb et al., 2003
	*LoGlyR*	R: 5′‐CCT GCT TGG AAG GCA AGT GTA CT‐3′	Serb et al., 2003
	*nadh1‐F*	F: 5′‐TGG CAG AAA AGT GCA TCA GAT TTA AGC‐3′	Buhay et al., 2002, Serb & Lydeard, 2003
	*nadh1‐R*	R: 5′‐GCT ATT AGT AGG TCG TAT CG‐3′	Buhay et al., 2002, Serb & Lydeard, 2003
*COI*	*LCO1490*	F: 5′‐GGT CAA CAA ATC ATA AAG ATA TTG G‐3′	Folmer et al., 1994
	*CO1F*	F: 5′‐GTT CCA CAA ATC ATA AGG ATA TTG G‐3′	Campbell et al., 2005
	*HCO700dy2*	R: 5′‐TCA GGG TGA CCA AAA AAY CA‐3′	Walker et al., 2006
*ITS1*	*18S*	F: 5′‐AAA AAG CTT CCG TAG GTG AAC CTG CG‐3′	King et al., 1999
	*5.8S*	R: 5′‐AGC TTG CTG CGT TCT TCA TCG‐3′	King et al., 1999

Two slightly different forward primers were used to amplify *COI* sequences for all species (Table 1). Sequences for *COI* were obtained for mussel specimens of *F. flava*, *F. subrotunda*, *P. cordatum*, and *P. rubrum* and *P. sintoxia* using the primers *LCO1490* (Folmer et al., 1994) and *HCO700dy2* (Walker et al., 2006). In the case of *P. plenum*, most sequences were obtained by using primers *COIF* (Campbell et al., 2005) and *HCO700dy2*. Details regarding PCR amplification appear in Supplemental Material S1.

We amplified the nuclear ribosomal internal transcribed spacer region subunit 1 (*ITS1*) sequence for mussel specimens molecularly identified as *P. rubrum* and *P. sintoxia* (with mitochondrial *COI* and/or *ND1* markers) from the Green River (40 mussel specimens). Sequences used as outgroups included *P. sintoxia* or *P. rubrum* from the Clinch River (four sequences) and Tennessee River (three sequences). Finally, two mussel specimens of *F. flava*, three *F*. *subrotunda*, two *P*. *cordatum*, and three *P*. *plenum* were sequenced and used as outgroups. Details regarding PCR amplification appear in Supplemental Material S1.

### 2.3 Data analysis

The consensus DNA sequences were obtained using Geneious® 7.0.6 (Biomatters, Inc.) and aligned using GeneStudio Version 2.2.0.0 (GeneStudio, Inc.). DNA sequence variation metrics—such as polymorphic nucleotide sites, number of haplotypes, nucleotide diversity, and haplotype diversity—were calculated using DnaSP 5.10 (Rozas et al., 2009). Pairwise difference values within and between species were estimated using *p*‐distances, and the most likely model of nucleotide substitution was identified using MEGA6 (Tamura et al., 2013).

For construction of phylogenetic trees and networks, the most appropriate model of nucleotide substitution was selected using MrModeltest 2 (Nylander, 2008), which works in an interface with PAUP 4.0 (Swofford, 1998). We selected the model with the lowest Akaike Information Criterion (*AIC*). For *COI*, *ND1*, and combined *COI + ND1* DNA sequences, the most appropriate model was the General Time Reversible (GTR + G + I) model with sites following a gamma distribution with a proportion of invariable sites. Due to the presence of gaps caused by nucleotide insertions and deletions (indels) in the sequence alignments for the *ITS1* region, we performed DNA sequence alignment using both the ClustalW (Thompson et al., 2003) plug‐in implemented in Geneious® 7.0.6 (Biomatters, Inc.) and also webPRANK (Löytynoja & Goldman, 2010). After alignment, FastGap v1.2 (Borchsenius, 2009) was used to encode observed indels. The most appropriate model was the symmetrical model (SYM + G) with gamma rates.

Phylogenetic trees were constructed using MrBayes 3.2.6 (Ronquist et al., 2012), and final trees were visualized using FigTree v1.4.2 (Rambaut, 2014). To analyze the MCMC runs resulting from MrBayes, we used Tracer v 1.6.0 (Rambaut et al., 2009). In this software, the effective sample size (*ESS*) was >200 for all the trees. Phylogenetic trees were constructed for each mitochondrial marker *COI* and *ND1*, using sequences from all the mussel specimens collected, and an additional tree was constructed using all markers combined. In addition, a phylogenetic tree for *P. sintoxia* and *P. rubrum* was constructed for the nuclear marker *ITS1*. These analyses incorporated four MCMC chains with trees sampled every 1000 generations *ND1* and combined *COI + ND1* and every 250 generations for *COI*. Finally, the *ITS1* trees were sampled every 100 generations. Species differentiation was assessed using the Automatic Barcode Gap Discovery (ABGD) method (Puillandre et al., 2012) using *ND1*, *COI*, and combined *COI + ND1* sequences. For *COI*, *ND1*, and combined *COI* + *ND1* sequences, species delimitation was assessed by using ABGD. To assign mussel specimens to species, the Kimura (1980) two‐parameter (K2P) distance model was used, where the minimum intraspecific genetic distance (*P*_min_) was set to 0.001 and the maximum intraspecific genetic distance (*P*_max_) was set to 0.1.

A phylogenetic network was constructed using SplitsTree4 (Huson & Bryant, 2006) for *COI* and *ND1* sequences. For both *COI* and *ND1* sequences, we included additional sequences from other species. To construct the network, we used the most appropriate evolutionary model to calculate the distances and the NeighborNet algorithm (Bryant & Moulton, 2002) for distance transformation.

For construction of the *COI* tree and split network, we added DNA sequences reported by Inoue et al. (2018), The species added included *Fusconaia askewi*, *F. cerina*, *F. chunii*, *F. flava*, *F. lanensis*, *F. masoni*, *F. subrotunda*, *Pleurobema cordatum*, *P. plenum*, *P. riddellii*, *P. rubrum*, and *P. sintoxia*. Finally, a sequence of *Pleuronaia dolabelloides* was added as an outgroup for the phylogenetic tree. These sequences were trimmed to match the length of the sequences for the phylogenetic analysis, resulting in many of them then being grouped into new haplotypes. Only one of these sequences for each new haplotype was added into the analysis used to reconstruct the phylogenetic tree. The added sequences as well as similar sequences, GenBank accession numbers and sampling locations for these added sequences are shown in Table S1. In addition, for construction of the *ND1* tree and split network, we added sequences for *Fusconaia askewi*, *F. lanensis*, *F. masoni*, *F. subrotunda*, *Pleurobema cordatum*, *P. plenum*, *P. rubrum*, and *P. sintoxia*. In addition, we added a sequence of *Pleurobema dolabelloides* as an outgroup.

We constructed separate *ITS* trees using the Clustal and webPRANK sequence alignments. Outgroup taxa for the *ITS1* analyses were collected in the Green River and included *F. flava* (*ITS1_Ffla*), *F. subrotunda* (*ITS1_Fsub*), *P. cordatum*, (*ITS1_Pcor*), and *P. plenum* (*ITS1_Pple*).

## 3 RESULTS

### 3.1 Amplification of molecular markers

We were able to PCR‐amplify *COI* using one reverse primer (HCO700dy2), but we needed two different forward primers (Table 1). The first forward primer, LCO1490 (Folmer et al., 1994), developed for a borad range of invertebrates, amplified *COI* sequences for individuals belonging to *F. flava*, *F. subrotunda*, *P. cordatum*, and *P. sintoxia/rubrum*. To amplify *COI* sequences for individuals subsequently identified as *P. plenum*, we used forward primer COIF (Campbell et al., 2005) that was developed for species in the tribe Pleurobemini (Burlakova et al., 2012; Campbell et al., 2005; Campbell & Lydeard, 2012a, 2012b).

We used two pairs of primers to amplify a 744‐bp region of *ND1*. The first primer set, *Leu‐uurF* and *LoGlyR* (Serb et al., 2003), which amplified sequences for individuals of *F. flava*, *F. subrotunda*, *P. cordatum*, and *P. plenum*, has been used successfully for a wide range of freshwater mussels (Schilling, 2015; Serb et al., 2003; Smith et al., 2018). However, we needed additional primers to amplify *ND1* sequences of *P. sintoxia* and *P. rubrum*, nadh1‐F, and nadh1‐F (Buhay et al., 2002; Serb & Lydeard, 2003), which have been used in other studies of mussels of Tribe Pleurobemini (Burlakova et al., 2012; Campbell & Lydeard, 2012a, 2012b; Campbell et al., 2008).

Nuclear *ITS1* sequences were amplified using primers 18S and 5.8S (King et al., 1999), which worked for most individuals of *P. sintoxia/rubrum* and for outgroups *F. flava*, *F. subrotunda*, *P. cordatum*, and *P. plenum*. A problem observed when amplifying these sequences was gaps among aligned sequences, which many times were due to an artifact of sequence quality. To ensure the quality of the sequences, we re‐sequenced those samples that presented extra bases and those not of high quality (<80% GC). Like Schilling (2015), we did not encounter length differences among sequences from the same mussel specimens. Hence, length differences were not quantified, as in Schilling (2015). The *ITS1* sequences used in this study included only one *ITS1* sequence for all individuals analyzed.

### 3.2 Haplotypes and variable sites

Haplotypes, GenBank accession numbers, and collection sites for all *COI*, *ND1*, and *ITS1* sequences are listed in Tables S1 and S2. The DNA sequences for *COI*, typically about 471 bp in length, were obtained for all 258 mussels collected from the Green River. These sequences resulted in six variable sites among haplotypes of *F. flava*, 14 variable sites for *F. subrotunda*, 46 for *P. cordatum*, 11 for *P. plenum*, and 21 for *P. sintoxia* and *P. rubrum*. Analysis of *COI* sequences resulted in 43 *F. flava* individuals presenting seven haplotypes, 22 *F. subrotunda* presenting 13 haplotypes, 117 *P. cordatum* presenting 43 haplotypes, 33 *P. plenum* presenting eight haplotypes, and 43 *P. sintoxia* and *P. rubrum* presenting 16 haplotypes. Mussel specimens collected from the Clinch (17 individuals) and Tennessee (5 individuals) rivers resulted in nine additional haplotypes for *P. plenum* and three additional haplotypes for *P. sintoxia* and *P. rubrum*.

Sequences for *ND1* typically were 744 bp, resulting in 14 variable sites among haplotypes of *F. flava*, 35 for *F. subrotunda*, 83 for *P. cordatum*, 12 for *P. plenum*, and 37 for *P. sintoxia* and *P. rubrum*. Analysis of *ND1* sequences resulted in 42 *F. flava* presenting 13 haplotypes, 20 *F. subrotunda* presenting 17 haplotypes, 116 *P. cordatum* presenting 58 haplotypes, 32 *P. plenum* presenting 12 haplotypes, and 41 *P. sintoxia* and *P. rubrum* collectively presenting 17 haplotypes. Some haplotypes from the respective species collected in the Green River were shared by mussels of those species collected from the Clinch and Tennessee rivers. However, numerous additional haplotypes were observed; for *F. subrotunda* one additional haplotype, *P. plenum* nine haplotypes, and *P. sintoxia* and *P. rubrum* four haplotypes.

The differing sample sizes for *ND1* and *COI* are due in part to not all the samples supporting amplification for the two markers. Combining the DNA sequences of the *COI* and *ND1* genes resulted in a 1215‐bp sequence, resulting in 15 haplotypic combinations for *F. flava*, 19 for *F. subrotunda*, 76 for *P. cordatum*, 15 for *P. plenum*, and 26 for *P. sintoxia* and *P. rubrum*, collectively. Mussel specimens collected from the Clinch and Tennessee rivers resulted in one additional haplotype for *F. subrotunda*, 13 for *P. plenum*, and five for *P. sintoxia* and *P. rubrum*.

For the nuclear *ITS1* DNA sequences, both the Clustal and WebPrank alignments resulted in a 448‐bp sequence containing five variable sites, which included three encoded gaps in both alignments. However, the positions of the encoded gaps differed between the two alignments. Five haplotypes were observed for *P. sintoxia* and *P. rubrum* (*ITS1_Psr03* to *ITS1_Psr07*) in the Green River samples, and two additional haplotypes were observed in samples from the Clinch and the Tennessee rivers (*ITS1_Psr01* to *ITS1_Psr02*). In addition, ten mussel specimens of other species from the Green River were used as outgroups in the phylogenetic analysis, including two *F. flava* (*ITS1_Ffla01* to *ITS1_Ffla02*), three *F. subrotunda* (*ITS1_Fsub01* to *ITS1_Fsub03*), two *P. cordatum* (*ITS1_Pcor01* to *ITS1_Pcor02*), and three *P. plenum* (*ITS1_Pple01* to *ITS1_Pple03*).

### 3.3 Genetic diversity

For *F. flava*, *F*. *subrotunda*, and *P. cordatum* observed haplotype and nucleotide diversities were higher for the *ND1* DNA sequences compared to the *COI* DNA sequences (Table 2). For *P. plenum* and *P. sintoxia/rubrum*, haplotype diversity was higher for *ND1* sequences, while nucleotide diversity was higher for *COI* sequences.

**TABLE 2:** Intraspecific variation of the mitochondrial DNA *COI* and *ND1* genes for species in the genera *Fusconaia* and *Pleurobema*.

Species	*N*	Number of variable sites	Number of haplotypes	Average number of nucleotide differences *k* (range)	Haplotype diversity (*h*)	Nucleotide diversity (*π*)
*COI*						
*Fusconaia flava*	43	6	7	0.368	0.339	0.00078
*Fusconaia subrotunda*	22	14	13	3.805	0.896	0.00808
*Pleurobema cordatum*	117	45	43	1.991	0.767	0.00423
*Pleurobema plenum*	33	11	8	2.655	0.701	0.00564
*Pleurobema sintoxia/rubrum*	43	21	16	1.797	0.85	0.00382
*ND1*						
*Fusconaia flava*	42	14	13	2.072	0.875	0.00278
*Fusconaia subrotunda*	20	35	17	6.595	0.984	0.00886
*Pleurobema cordatum*	116	54	58	3.724	0.93	0.00501
*Pleurobema plenum*	32	17	12	2.738	0.768	0.00368
*Pleurobema sintoxia/rubrum*	41	37	18	2.756	0.89	0.0037

*Note*: Mussel specimens were collected in 2015 and 2017 from Pool 4 (GPS coordinates = 37.18286, −86.6296; river mile = 149) and Mammoth Cave National Park (GPS coordinates 37.17819, −86.1154; river mile = 197) in the Green River, Kentucky.

For *COI* sequences, the species with the highest haplotype diversity was *F. subrotunda* (0.896), followed by *P. sintoxia/rubrum* (0.850), *P. cordatum* (0.767), *P. plenum* (0.701), and *F. flava* (0.339) (Table 2). Nucleotide diversity was highest in *F. subrotunda* (0.00808), followed by *P. plenum* (0.00564), *P. cordatum* (0.00423), *P. sintoxia/rubrum* (0.00382), and *F. flava* (0.00078). For *ND1* sequences, the species with the highest haplotype diversity was *F. subrotunda* (0.984), followed by *P. cordatum* (0.930), *P. sintoxia/rubrum* (0.890), *F. flava* (0.875), and *P. plenum* (0.768). The nucleotide diversity was highest in *F. subrotunda* (0.00886), followed by *P. cordatum* (0.00501), *P. sintoxia/rubrum* (0.00370), *P. plenum* (0.00368), and *F. flava* (0.00278). Among the species investigated, *P. cordatum* exhibited the highest haplotype diversities at both *COI* (0.77) and at *ND1* (0.93) (Table 2). These values for *ND1* were slightly lower than those reported by Jones et al. (2015) in the Green River, KY (0.97) and Tennessee River, TN (1.0). Their sample sizes, 18 mussel specimens for both rivers, were considerably smaller than sample sizes in this study. In our results, nucleotide diversity for *P. cordatum* ranged from 0.004 at *COI* and 0.005 at *ND1*, slightly higher than values reported by Jones et al. (2015), between 0.003 in the Green River, KY and 0.00361 in the Tennessee River, TN. For *P. plenum*, our nucleotide diversity ranged between 0.00368 and 0.00564 for *ND1* and *COI*, respectively. This was comparable to the values reported by Jones et al. (2015), in which *ND1* nucleotide diversity was between 0.003 and 0.005 for the Green River, KY and the Clinch River, TN, respectively.

### 3.4 Phylogenetic analysis

Phylogenetic trees of DNA sequence haplotypes were constructed to visualize relationships among the respective lineages. The topology of the *COI* tree, showing species identifications and clades (Olivera‐Hyde, 2021), was consistent with that of Inoue et al. (2018). In addition, haplotypes for each species collected from the Green River consistently grouped into the same species clades as haplotypes from specimens collected in the Clinch and Tennessee rivers, including one *F. subrotunda* (collected from the Clinch River), 14 *P. plenum* (12 from the Clinch River and two from the Tennessee River), and seven *P. sintoxia* and *P. rubrum* (four from the Clinch River and three from the Tennessee River). The additional sequences of *Fusconaia flava* grouped together with sequences of *F. flava* and *F. cerina* from Inoue et al. (2018). Our sequences grouped together with species of *Fusconaia askewi*, *F. cerina*, *F. chunni*, *F. lanensis*, and *F. masoni*. However, a lower prior maximal distance in ABGD (*P* = 1.00 e^−3^) resulted in the *F. flava* haplotypes grouping with the haplotypes of Inoue's for *F. flava* and with *F*. *cerina*. The Green River *F. flava* haplotypes were different from some of the *F. flava* obtained from Arkansas. In addition, our mussel specimens of *P. sintoxia* and *P. rubrum* grouped together in the same clade with those of the same species reported by Inoue et al. (2018); this is particularly interesting, as the authors added sequences from several locations where *P. rubrum* and *P. sintoxia* occur. The *P. sintoxia/rubrum* clade was paraphyletic with *Pleurobema riddelli*, which seems to be closely related.

In the *ND1* tree, sequences from our specimens grouped together with those from Bertram (2015), Burlakova et al. (2012), Jones et al. (2015), Marshall et al. (2018), and Schilling (2015). Well‐defined clades were observed for *F. subrotunda*, *P. cordatum*, *P. plenum*, and *P. sintoxia/rubrum*. The latter clade showed specimens from this and other studies grouping together, suggesting that *P. sintoxia* and *P. rubrum* are conspecific based on mtDNA (Figure 4). In the case of *F. flava*, an initial partition using ABGD resulted in *F. flava* haplotypes grouping together with *F. askewi*, *F. lanensis*, and *F. masoni*. However, a recursive partition separated the *F. flava* sequences from *F. askewi*, *F. lanensis*, and *F. masoni*. As in the *COI* tree, *F. askewi* and *F. lanensis* grouped together.

**FIGURE 5:**
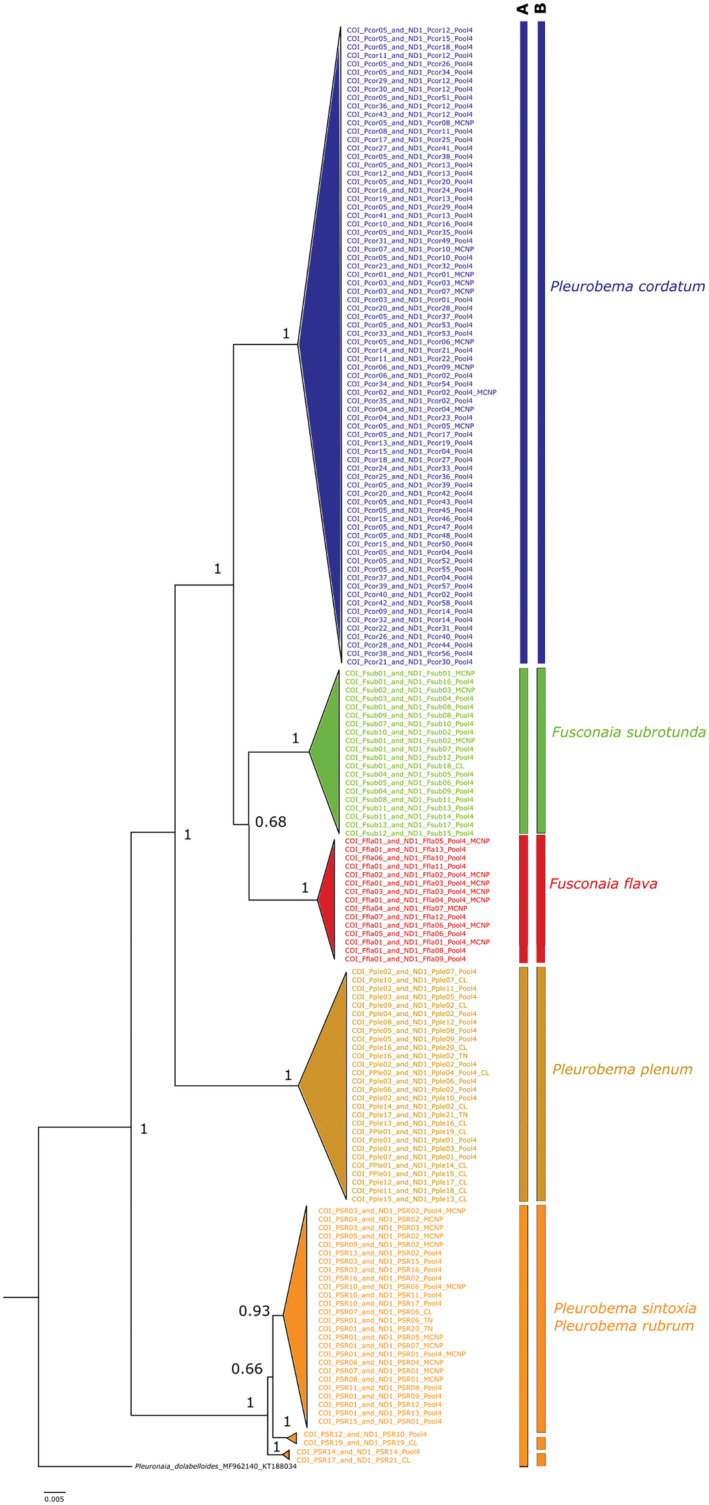
Phylogenetic tree constructed using mitochondrial *COI* + *ND1* genes sequences and Bayesian consensus trees in MrBayes. The most appropriate model of nucleotide substitution selected using the Akaike Information Criterion (*AIC*) was the General Time Reversible (GTR + G + I) model that followed a gamma distribution with a proportion of invariable sites. The analysis was run with 6 million generations, and trees were sampled every 1000 generations, which generated a total of 9002 trees. The final standard deviation of split frequencies was 0.009964 with a −ln likelihood of −5851.39. Posterior probabilities are indicated to the left of the respective nodes. The outgroup was *Pleuronaia dolabelloides* (MF962140 + KT188034). Species differentiation was assessed using the Automatic Barcode Gap Discovery (ABGD). To assign mussel specimens into the different hypothetical species, we used the Kimura (1980) two‐parameter (K2P) distance model, where the minimum intraspecific genetic distance (*P*_*min*_) was set as 0.001, and the maximum intraspecific genetic distance (*P*_*max*_) was set as 0.1. Two partitions for species delimitation, partition (A) has a prior maximal distance of P = 1.67 e^−3^, and partition B is its recursive partition with the same prior maximal distance. Mussel specimens were collected in 2015 and 2017 from Pool 4 (GPS coordinates = 37.18286, −86.6296; river mile = 149) and Mammoth Cave National Park (GPS coordinates 37.17819, −86.1154; river mile = 197) in the Green River, Kentucky, and additional mussel specimens were collected from the Clinch River (CL) and Tennessee River (TN).

The phylogenetic tree constructed from combined *COI* and *ND1* sequences resulted in five well‐defined clades that included *F. flava*, *F. subrotunda*, *P. cordatum*, *P. plenum*, and *P. sintoxia/rubrum* (Figure 5). As in the separate trees for *COI* and *ND1*, the tree of combined sequences showed little evidence to support these clades as different species for *P. rubrum* and *P. sintoxia*. There are four individuals in the *P. sintoxia/rubrum* clade that were separated in two additional clades when *COI* and *ND1* were combined and species delimitation was assessed. Two of these individuals shared the *ITS1*_*PSR03* (tag # WE779 from Pool 4, and tag # BLU009 from the Clinch River) sequence. The other two individuals, one from Pool 4 (tag # WG591) and another from the Clinch River (tag#RubClinch) have *ITS1_ PSR04* and *ITS1_PSR01*, respectively. Only one individual was identified as *P. rubrum* by the five mussel identification experts.

### 3.5 Split networks

**FIGURE 6:**
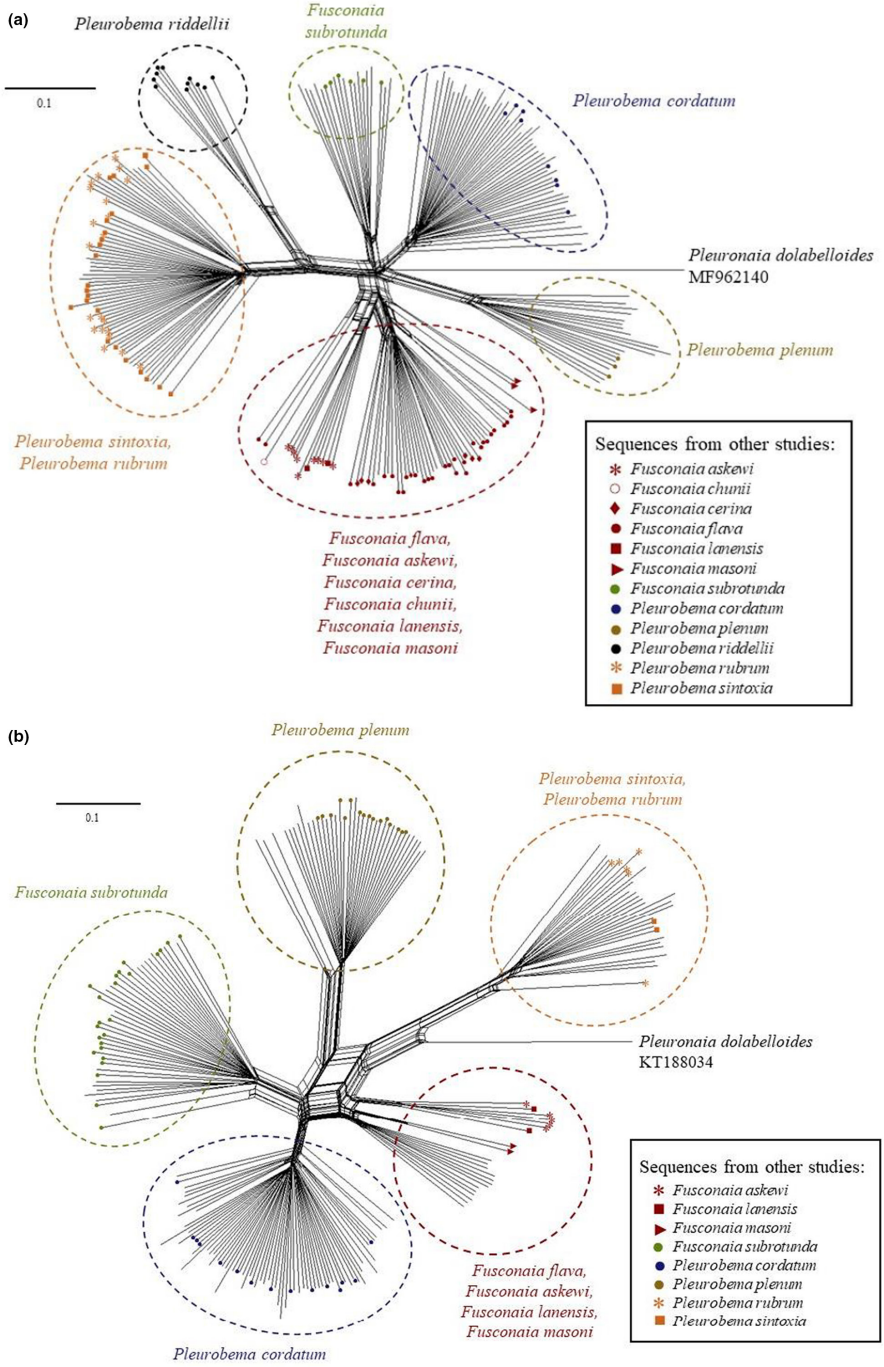
Split phylogenetic network using mitochondrial DNA *COI* gene (a) and *ND1* gene sequences (b). For both Split networks, distances were calculated using GTR with a gamma rates model and a proportion of invariable sites estimated with splits Tree4 (Huson & Bryant, 2006). The algorithm used for distances transformation was NeighborNet. Mussel specimens were collected in 2015 and 2017 from Pool 4 (GPS coordinates = 37.18286, −86.6296; river mile = 149) and Mammoth Cave National Park (GPS coordinates 37.17819, −86.1154; river mile = 197) in the Green River, Kentucky. Additional mussel specimens of *P. rubrum* were collected from the Clinch River, Hancock County, TN, and the Tennessee River downstream of Pickwick dam, Hardin County, TN. For COI, the reference sequences were obtained from Inoue et al., 2018 with the associated accession numbers available in Table S1. For ND1, reference sequences for *Fusconaia askewi*, *F. lanensis*, *F. masoni*, *F. subrotunda*, *Pleurobema cordatum*, *P. plenum*, and *P. sintoxia* were obtained from Bertram et al. 2015; Burlakova et al., 2012; Jones et al., 2015; Marshall et al., 2018; Schilling, 2015 with associated accession numbers available in Table S1. The outgroups are *Pleuronaia dolabelloides COI* sequence (GenBank accession number: MF962140) and *ND1* sequence (GenBank accession number: KT118034).

**FIGURE 7:**
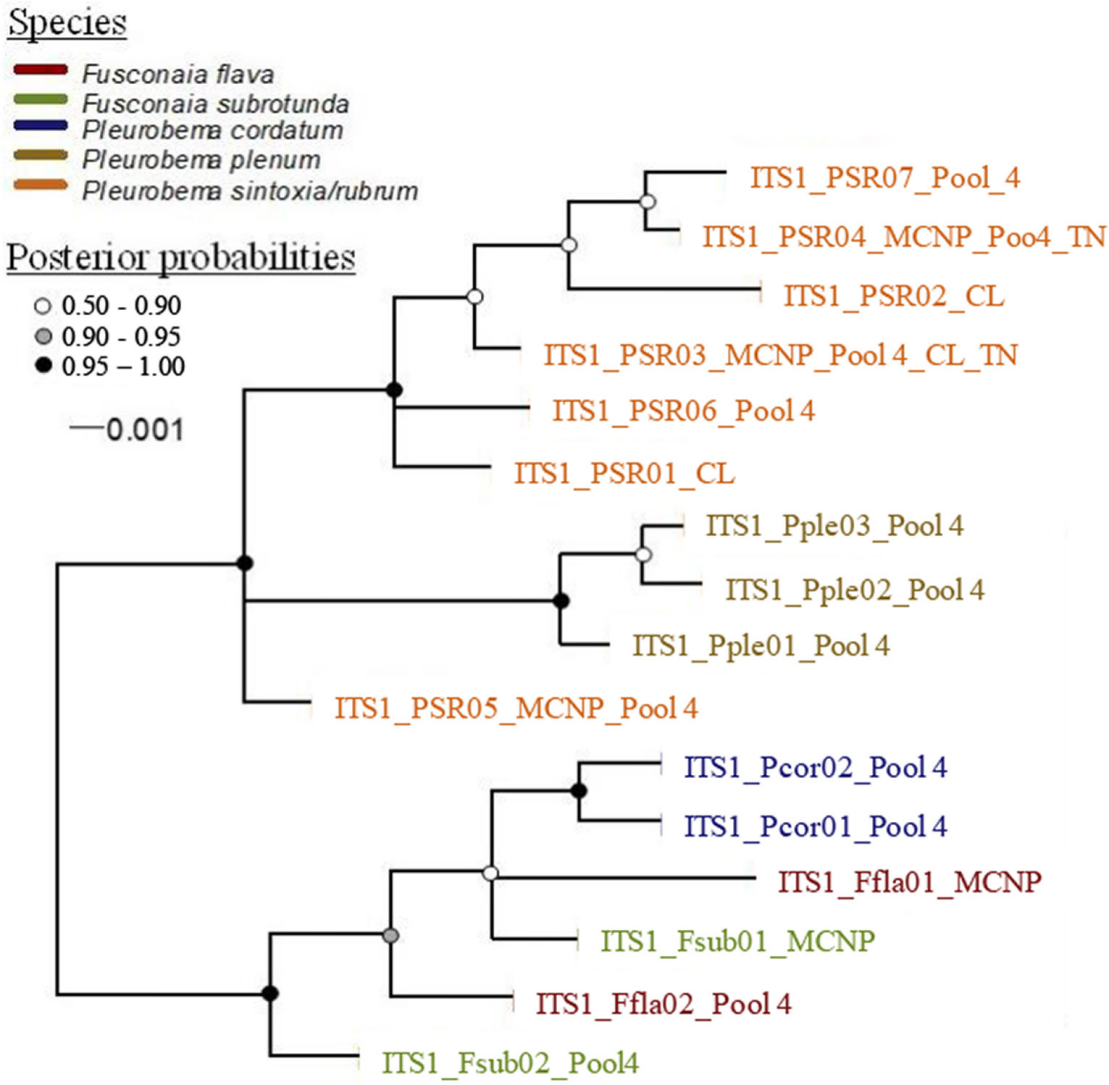
Phylogenetic trees constructed using *ITS1* sequences with clustal alignment and Bayesian consensus trees that were constructed using MrBayes. The most appropriate model of nucleotide substitution using the Akaike information criterion (*AIC*) was the symmetrical model (SYM + G) with gamma rates. The analysis was run with 400,000 generations, and trees were sampled every 100 generations, which generated a total of 1785 trees. The final standard deviation of split frequencies was lower than 0.01 with a −ln likelihood of −982.06. The outgroups that were collected from the Green River and included *F. flava* (*ITS1_Ffla*), *F. subrotunda* (*ITS1_Fsub*), *P. cordatum*, (*ITS1_Pcor*), and *P. plenum* (*ITS1_Pple*). Mussel specimens and outgroups were collected in 2015 and 2017 from Pool 4 (GPS coordinates = 37.18286, −86.6296; river mile = 149) and mammoth cave National Park (GPS coordinates 37.17819, −86.1154; river mile = 197) in the Green River, Kentucky. Additional outgroups were collected from the Clinch River (CL) and Tennessee River (TN).

Topologies of the split networks resulting from analysis of *COI*, *ND1*, and *COI + ND1* sequences were consistent and showed five distinct clades, *F. flava*, *F. subrotunda*, *P. cordatum*, *P. plenum*, and *P. sintoxia*/*rubrum*. In the *COI* split networks (Figure 6a), we added sequences of *P. sintoxia* and *P. rubrum* from Inoue et al. (2018), which clustered within our *P. sintoxia/rubrum* clade. The same results were observed when *ND1* sequences of *P. sintoxia* and *P. rubrum* from Jones et al. (2015) were included (Figure 6b). Split networks constructed from *ITS1* sequences included both *P. sintoxia* and *P. rubrum*, as well as outgroups from other species, such as *F. flava*, *F. subrotunda*, *P. cordatum*, and *P. plenum*. For both *ITS1* alignments, one of the haplotypes of *P. sintoxia/rubrum* (*ITS1_Psr05*) seemed to be particularly distinct from the other haplotypes in this clade. However, specimens from the same outgroup species fell into different clades, making inference of species‐level differentiation using *ITS1* sequences unreliable (Figure 7).

### 3.6 Pairwise differentiation

In the case of the mitochondrial *COI* gene, pairwise differentiation values using the Tamura‐Nei with invariable sites nucleotide model of mutation for the *COI* sequences ranged between 0.043 between *F. subrotunda* and *F. flava* and 0.091 between *P. plenum* and *P. sintoxia/rubrum*, whereas pairwise differentiation calculated using a *p*‐distance model among species ranged from 0.041 between *F. flava and F. subrotunda* to 0.083 between *P. sintoxia/rubrum* and *P. plenum*. The *p*‐distance pairwise differentiation within species was highest for *P. plenum* at 0.006, and lowest for *F. flava* at 0.001 (Table 3). In the case of *NDI*, pairwise differentiation values using the Tamura‐Nei with invariable sites model ranged from 0.050 between *F. subrotunda* and *F. flava* and 0.108 between *F. subrotunda* and *P. sintoxia/rubrum*, whereas pairwise differentiation calculated using *p*‐distances ranged from 0.046 between *F. flava* and *P. cordatum* to 0.098 between *P. cordatum* and *P. sintoxia/rubrum*. Pairwise differentiation within species was highest for *F. subrotunda* at 0.009, while the lowest value was estimated for *F. flava* at 0.003. The pairwise *p*‐distance results showed that mean intraspecific differentiation among individuals of *F. subrotunda* was 0.8% for *COI* and 0.9% for *ND1*, which were the highest intraspecific distances observed for the species studied in the Green River. These results are similar to those of Schilling (2015), who reported an *ND1* intraspecific difference of 1% among individuals of *F. subrotunda* in the upper Tennessee River basin. Higher intraspecific differences values for *F. subrotunda* were reported by Burlakova et al. (2012), in which *COI* pairwise differences ranged from 1.23% to 1.24%, while *ND1* pairwise differences ranged between 1.11% and 1.30%. Interspecific *COI* pairwise differences showed that the species most closely related to *F. subrotunda* was *F. flava* (4.1%), followed by *P. cordatum* (4.2%). These results were comparable to the *ND1* pairwise differences, 4.7% among *F. subrotunda* and both *F. flava* and *P. cordatum*. In addition, pairwise differences paralleled results from the phylogenetic trees, split networks, and haplotype networks constructed using *COI*, *ND1*, and *COI + ND1* sequences. Species differentiation tested with ABGD resulted in a well‐differentiated *F. subrotunda* clade.

**TABLE 3:** Estimates of evolutionary divergence based on analysis of mitochondrial DNA *COI* and *ND1* sequence pairs between and within species of *Fusconaia* and *Pleurobema* using *p*‐distances (lower diagonal) and the Tamura‐Nei with invariable sites model of nucleotide substitution (upper diagonal).

	*Fusconaia flava*	*Fusconaia subrotunda*	*Pleurobema cordatum*	*Pleurobema plenum*	*Pleurobema sintoxia/rubrum*
*COI*					
*Fusconaia flava*	**0.003**	0.05	0.05	0.089	0.098
*Fusconaia subrotunda*	0.047	**0.009**	0.051	0.089	0.108
*Pleurobema cordatum*	0.046	0.047	**0.005**	0.087	0.011
*Pleurobema plenum*	0.08	0.079	0.078	**0.004**	0.01
*Pleurobema sintoxia/rubrum*	0.086	0.094	0.098	0.088	**0.004**
*ND1*					
*Fusconaia flava*	**0.001**	0.043	0.053	0.074	0.077
*Fusconaia subrotunda*	0.041	**0.008**	0.044	0.069	0.07
*Pleurobema cordatum*	0.05	0.042	**0.004**	0.067	0.075
*Pleurobema plenum*	0.068	0.064	0.062	**0.006**	0.091
*Pleurobema sintoxia/rubrum*	0.071	0.064	0.069	0.083	**0.004**

*Note*: Bold numbers represent estimates within species. Mussel specimens were collected in 2015 and 2017 from Pool 4 (GPS coordinates = 37.18286, −86.6296; river mile = 149) and Mammoth Cave National Park (GPS coordinates 37.17819, −86.1154; river mile = 197) in the Green River, Kentucky.

Intraspecific pairwise *p‐*distances for mussel specimens of *P. cordatum* ranged between 0.4% for *COI* and 0.5% for *ND1* (Table 3). These results were similar to those reported by Jones et al. (2015), in which *ND1* intraspecific distances for *P. cordatum* ranged between 0.4% and 0.7%. Results for interspecific pairwise distances showed that the highest differences were between *P. cordatum* and *P. sintoxia/rubrum*, with 6.9% for *COI* and 9.8% for *ND1*. The second‐most differentiated species from *P. cordatum* was *P. plenum*, with 6.2% *p*‐distance for *COI* and 7.8% for *ND1*.

Intraspecific variation among mussel specimens of *P. plenum* ranged between 0.4% and 0.6% for *COI* and *ND1* sequences, respectively. These results were similar to those reported by Jones et al. (2015), who reported *ND1* intraspecific pairwise differences between 0.6% (Green River, Kentucky) and 0.8% (Tennessee River, Tennessee) for *P. plenum*. In this study, intraspecific pairwise differences were highest between *P. plenum* and *P. sintoxia/rubrum*, which were between 8.3% and 8.8% for *COI* and *ND1* sequences, respectively. Finally, intraspecific pairwise differences for *P. sintoxia/rubrum* were 0.4% for both *COI* and *ND1* sequences, comparable to those of Jones et al. (2015), who reported *ND1* intraspecific pairwise differences of 0.1% for *P. sintoxia* and 0.8% for *P. rubrum*.

## 4 DISCUSSION

Most mussel species show substantial differences in shell morphology and life history traits, and thus genetic differentiation among most species is high and easy to distinguish. However, species belonging to genera *Fusconaia* and *Pleurobema* are particularly difficult to identify morphologically, even by trained eyes. Our phylogenetic assessment of the *Fusconaia* and *Pleurobema* species in the Green River, KY resulted in the identification of five well‐differentiated clades (*F. flava*, *F. subrotunda*, *P. cordatum*, *P. plenum*, and *P. sintoxia/rubrum*). While our study neither uncovered cryptic species nor differentiate individuals of *P. rubrum* and *P. sintoxia* as separate species, it showed high observed haplotype and nucleotide diversity within populations, suggesting that these investigated populations are large and genetically diverse in the Green River.

### 4.1 Molecular markers

The concept of using DNA sequences to “barcode” species relies on intraspecific variation being clearly lower than interspecific variation for mitochondrial (*COI*, *ND1*) and nuclear markers (*ITS1*). The most‐used marker for DNA barcoding in eukaryotes is the mitochondrial *COI* gene (Bleidorn, 2017). The principal practical reasons for use of mitochondrial markers for barcoding are the availability of “universal” PCR primers, larger numbers of mtDNA copies per cell relative to nuclear DNA, and high interspecific variation that gives rise to so‐called barcoding “gaps” (Puillandre et al., 2012). However, the use of only mitochondrial markers in phylogenetic studies has been criticized due to their solely maternal mode of inheritance, inconsistent mutation rate, limited power to detect introgression, low effective population size, low information content among closely related species, and heteroplasmy or pseudogenization (Bleidorn, 2017). In this study, for *COI* and *ND1* primers, target DNA from some specimens of *P. plenum* and *P. sintoxia/rubrum* may have amplified or not with one primer‐pair combination due to DNA sequence variation at the primer‐binding site.

The principal limitations for the *ITS1* marker were low nucleaotide variability and too few fixed nucleotide mutational states to distinguish some of the study species. Species delimitation using AGBD was not possible for *ITS1* sequences. The *ITS1* marker split the sequences into too many groups, erratically mixing species that were well defined with the mitochondrial markers; e.g., over‐splitting occurred for sequences of *F. flava* and *P. cordatum*, as well as *P. sintoxia/rubrum*. Over‐splitting could be due to higher intraspecific variation relative to interspecific variation. The sequencing of this marker is challenging because each individual inherits two copies from the parents. Other challenges for the *ITS1* marker are the presence of gaps in the aligned sequences. The number of nucleotide differences was not high enough for successful use of species delimitation using ABGD. The two haplotype sequences for *F. flava* (*ITS1_Ffla01* and *ITS1_Ffla02*) and *F. subrotunda* (*ITS1_Fsub01* and *ITS1_Fsub02*) came from two different sampling locations (Pool4 and MCNP), which could explain the oversplitting within these two species. However, the data for these two species are too limited to conclude that *ITS1* sequences are appropriate for population‐level studies. The results from Elderkin (2009) suggested the contrary; amplified *ITS1* sequences from *Cumberlandia monodonta* individuals showed higher genetic diversity within individuals due to heterozygosity than genetic variation among individuals. In addition, separation of haplotypes of *F. flava* (*F*_ST_ = −0.019, *p* = .776) and *F. subrotunda* (*F*_ST_ = 0.077, *p* = .077) per population is not supported by genetic differentiation analysis using combined *COI* + *ND1* sequences that suggested that individuals from Pool 4 and MCNP belong to the same population (Olivera‐Hyde, 2021). Our results for the *ITS1* marker matched those of Schilling (2015), who observed that some estimates for interspecific variation were lower than those for intraspecific variation.

### 4.2 Phylogenetic assessment

Delineation of species in the genera *Fusconaia* and *Pleurobema* is supported by a suite of morphological and life‐history traits (Olivera‐Hyde, 2021). In the Green River, a principal morphological difference which generally is reliable is foot color. Mussels in the genus *Pleurobema* typically have a white foot, whereas mussels in the genus *Fusconaia* typically have an orange foot. Two important characters for identification of *Fusconaia* and *Pleurobema* species are conglutinate shape and the number of gills used to brood eggs and larvae. For *Fusconaia* species, conglutinates are slender and subcylindrical and all four gills are used to brood eggs and larvae, whereas for *Pleurobema* conglutinates are leaf‐like and can have different layers and only the outer pair of gills are used to brood eggs and larvae (Barnhart et al., 2008; Haag & Warren, 2003). The two *Fusconaia* species in the Green River were characterized by distinct shell shapes, with *F. flava* being trapezoidal and *F. subrotunda* being more rounded and elongate.

### 4.3 Pyramid and round pigtoes

In this study, we collected a relatively large number of individuals identified morphologically as *P. sintoxia* and *P. rubrum* from the Green River, KY to enhance the probability of delineating these two nominal taxa and for detecting any cryptic species which could have small populations and prove similar in appearance to these two species. By using a large sample size, any intraspecific and interspecific nucleotide differences are more detectable and better characterized, enabling identification of any species‐level differences among taxa. Further, we added DNA sequences of *P. sintoxia* and *P. rubrum* from other studies, to include *COI* (Inoue et al., 2018) and *ND1* (Jones et al., 2015), and all these sequences grouped together phylogenetically. Thus, even when utilizing all these DNA sequences, we did not observe clear differentiation between nominal *P. rubrum* and *P. sintoxia*. When forcing the species delimitation for a higher species separation, we observed the formation of not two, but rather three clades when screening *COI* and *ND1* sequences. One of the individuals exhibited haplotype *COI_PSR14_and_ND1_PSR14* and was the only one consistently identified as *P. rubrum* by the experts, and the other individual was identified as *P. sintoxia* by three of four experts. The *ITS1* sequences for these individuals were *ITS1_PSR3* and *ITS1_PSR4*, respectively. However, this phylogenetic separation was not corroborated by results of the other phylogenetic analysis methods applied, such as construction of haplotype networks and split networks. Morphologically, individuals of *P. sintoxia* and *P. rubrum* can look very distinct from each other, especially regarding shell shapes (Figure 1). For *P. rubrum*, the shell shape is typically a scalene triangle with beaks facing forward and with a very marked sulcus that traverses the shell from near the umbo to the ventral margin, whereas for *P. sintoxia*, individuals are much more rounded in shape with beaks facing each other, and without a well‐defined sulcus. Separation of these two putative species seems to be supported by morphological differences in their glochidia (Culp et al., 2009). Thus, it is important to investigate quantitatively whether glochidial differences exist between mussels expressing the *P. rubrum* and *P. sintoxia* shell forms. Additional lines of evidence to test species boundaries between these two nominal taxa should include phylogenomic studies, which could test for potential phylogenetic differentiation at the genome level. In comparison to traditional molecular phylogenetics which is conducted by Sanger sequencing, phylogenomics uses much larger amounts of DNA sequence data, which produces large number of single nucleotide polymorphism (SNP) markers and thereby reduces sampling error. Hence, the use of phylogenomics presumably would result in better taxonomic resolution. Another line of evidence would be analysis of how the shape and size of the shell of *P. sintoxia* and *P. rubrum* changes morphologically with changes in stream size from small to large river systems. Known as Ortmann's (1920) law of stream position, such morphological changes have been described for *F. flava*, for example, where the shell inflation index (width/length × 100) increases from small streams to large rivers (Haag, 2012).

Hybridization and backcrossing between individuals of *P. rubrum* and *P. sintoxia* may have resulted in introgression of the mtDNA genome between these species, making them indistinguishable at this marker. Assuming that these taxa represent distinct species in the Green River, hybridization may have resulted in introgression of mtDNA from one species to the other. Thus, interpretation of this data should be constrained to the individuals collected from the Green River, and such hybridization and introgression processes should be tested for in the different drainages where these two putative species occur sympatrically. There are other examples in the genus *Pleurobema* of species that are phylogenetically indistinguishable at mtDNA but are morphologically distinct, such as *P. clava* and headwater populations of *P. oviforme* (Morrison et al., 2021).

### 4.4 Genetic diversity

Haplotype diversities in our study were high, suggesting that this high contemporary genetic diversity could be due to these species historically occurring in much larger, interconnected populations that were linked to those in the mainstem of the Ohio River and its nearby tributaries. That is, the high contemporary genetic diversity in the Green River could be the result of this demographic signal still being maintained in these populations. Impoundments have driven decline of species such as *P. clava* and many other pigtoe species throughout the Ohio River system (Haag & Cicerello, 2016), as many of these species are intolerant of the altered flow conditions. However, other species such as *P. cordatum* seem more tolerant of impoundments and may adapt to altered riverine systems. In contrast, *P. plenum* seems minimally tolerant of impoundments, and thus much of its historically suitable habitat throughout the Ohio River system has been altered or destroyed by dams. Both *P. rubrum* and *P. sintoxia* are at best marginally tolerant to impounded stream conditions. Future studies should assess the effect of impoundment on shell shape variation among individuals of *P. sintoxia/rubrum*. Individuals of *F. flava* seem able to adapt to a variety of habitats, and its populations are generally stable throughout the species' range, whereas *F. subrotunda* prefers unimpounded large stream environments and is declining throughout its range. Assessment of recruitment is important for all of these species, as unsuitable water quality and altered hydrology can decrease or even halt reproduction, and hence the high haplotype diversity that we observed could be a measure of old, nonrecruiting and demographically imperiled populations. Recruitment failure in these populations would be catastrophic, as the extirpation of these populations from the Green River would have serious consequences for the long‐term conservation of these species. Fortunately, there is evidence of recruitment for all five investigated species in the Green River, which is one of the best refuge strongholds for these and many other species in the Ohio River system (Haag & Cicerello, 2016). Periodic monitoring to assess the abundance, recruitment, and genetic diversity of the Green River mussel fauna will be critical for managing the viabilty of these species.

### 4.5 Management implications

The IUCN Red List status for *F. flava* is “least concern,” although this species showed the lowest haplotype and nucleotide diversity among our study species. The effective population size (*N*_e_) has not been estimated for this species, mainly due to a lack of PCR primers for DNA microsatellites specifically designed for this species or even for a closely related *Fusconaia* species. The *Fusconaia subrotunda* clade was well supported phylogenetically and was the clade with the highest nucleotide and haplotype diversities in the study. Principal concerns regarding management of these species include continued demographic declines as large‐ to medium‐sized free‐flowing riverine habitats are lost (Haag & Cicerello, 2016). Future efforts are needed to develop nuclear DNA genetic markers to estimate *N*_e_ and quantify genetic diversity of this species in the Green River.

*Pleurobema cordatum* numbers have declined range‐wide, likely due to the reduction of large river habitats. However, *P. cordatum* seems more tolerant of impoundments than *F. subrotunda* and other *Pleurobema* species (Haag & Cicerello, 2016). This species showed higher nucleotide diversity (*π*) and smaller haplotype diversities (*h*) than other *P. cordatum* populations reported for the Green and the Tennessee Rivers (Jones et al., 2015).

The federally protected *Pleurobema plenum* has been listed under the U.S. Endangered Species Act as endangered since 1976 and its recovery plan was approved in 1984 (U.S. Fish and Wildlife Service, 1976, 1984). *Pleurobema plenum* is not very tolerant of impoundments and has been extirpated from most of its historical range. Because *P. plenum* is sensitive to habitat modification, its critical habitat (medium‐ to large‐sized rivers) must be protected. However, high haplotype diversity suggests that the *P. plenum* population is healthy and reasonably abundant in the Green River, KY. Recruitment and abundance of this species needs to be regularly monitored to ensure these values are not indicative of an aging, potentially nonrecruiting population.

While both *P. sintoxia* and *P. rubrum* appear to belong to only one phylogenetic clade based primarily on mtDNA, these two nominal taxa should be treated as separate species until additional morphological and nuclear DNA marker‐based studies have been completed. Similar to most of the species collected from the Green River, specimens belonging to the *P. sintoxia/rubrum* clade seem to be marginally tolerant to even intolerant of impounded riverine conditions (Haag & Cicerello, 2016). Ongoing studies assessing morphological differentiation between these two putative species are being conducted by Dr. Monte McGregor at the Center for Mollusk Conservation of the Kentucky Wildlife Resources Agency, who is currently assessing glochidial morphological differences.

Finally, in contrast to Schilling (2015), who performed a similar molecular marker‐ and morphology‐based study of mussels in the Tennessee River basin and found evidence of several cryptic species in the genus *Pleurobema* and *Pleuronia*, we did not find cryptic species in the Green River. An additional species that has been reported for the Green River, the endangered clubshell (*P. clava*) (Haag & Cicerello, 2016), still occurs in the river upstream of the sampling sites, but was not found during the field collections in Pool 4 and MCNP. This species was reported from the Green River (Kentucky) in Hart and Taylor counties by Watters (1994). However, he did not find live mussels, but only fresh‐dead shells. Thus, future studies are needed to monitor the recruitment, abundance, and genetic diversity of this species there to determine population status.

## AUTHOR CONTRIBUTIONS

**Eric M. Hallerman:** Conceptualization (equal); funding acquisition (equal); investigation (equal); project administration (equal); resources (equal); supervision (equal); writing – review and editing (equal). **Jess W. Jones:** Conceptualization (equal). **Miluska Olivera‐Hyde:** Conceptualization (equal); data curation (equal); formal analysis (equal); investigation (equal); methodology (equal); validation (equal); visualization (equal); writing – original draft (equal); writing – review and editing (equal).

## CONFLICT OF INTEREST

None declared.

## Supporting information


Appendix S1.

## Data Availability

GenBank accession numbers for the haplotype sequences are listed in Table S1.

## References

[ece39717-bib-0002] Barnhart, M. C. , Haag, W. R. , & Roston, W. N. (2008). Adaptations to host infection and larval parasitism in Unionoida. Journal of the North American Benthological Society, 27, 370–394.

[ece39717-bib-0003] Bertram, E. P. (2015). Confirmation of potential cyprinid hosts for a state threatened freshwater mussel of East Texas . [M.S. Thesis, University of Texas at Tyler]. https://scholarworks.uttyler.edu/cgi/viewcontent.cgi?referer=https://scholar.google.com/&httpsredir=1&article=1022&context=biology_grad

[ece39717-bib-0004] Bleidorn, C. (2017). Phylogenomics (pp. 35–36). Springer International Publishing.

[ece39717-bib-0005] Bogan, A. E. (1996a). *Pleurobema plenum*. The IUCN red list of threatened species 1996: E.T17669A7284466. 10.2305/IUCN.UK.1996.RLTS.T17669A7284466.en

[ece39717-bib-0006] Bogan, A. E. (1996b). *Pleurobema clava*. The IUCN red list of threatened species 1996: E.T17665A7280212. 10.2305/IUCN.UK.1996.RLTS.T17665A7280212.en

[ece39717-bib-0007] Bogan, A. E. (1996c). *Pleurobema rubrum*. The IUCN red list of threatened species 1996: E.T17670A7286106. 10.2305/IUCN.UK.1996.RLTS.T17670A7286106.en

[ece39717-bib-0008] Bogan, A. E. , & Seddon, M. B. (1996). *Pleurobema cordatum*. The IUCN red list of threatened species 1996: E.T17678A7303141. 10.2305/IUCN.UK.1996.RLTS.T17678A7303141.en

[ece39717-bib-0009] Borchsenius, F. (2009). FastGap 1.2. Department of Biological Sciences. University of Aarhus. https://www.aubot.dk/FastGap_home.htm

[ece39717-bib-0010] Bryant, D. , & Moulton, V. (2002). *NeighborNet: An agglomerative method for the construction of planar phylogenetic networks*. Workshop on algorithms in bioinformatics 2002. Lecture Notes in Computer Science, vol. 2452. (Springer, Berlin, Heidelberg). 10.1007/3-540-45784-4

[ece39717-bib-0011] Buhay, J. E. , Serb, J. M. , Dean, C. R. , Parham, Q. , & Lydeard, C. (2002). Conservation genetics of two endangered unionid bivalve species, *Epioblasma florentina walkeri* and *E*. *capsaeformis* (Unionidae: Lampsilini). Journal of Molluscan Studies, 68, 385–391.

[ece39717-bib-0012] Burlakova, L. E. , Campbell, D. , Karatayev, A. Y. , & Barclay, D. (2012). Distribution, genetic analysis and conservation priorities for rare Texas freshwater molluscs in the genera *Fusconaia* and *Pleurobema* (Bivalvia: Unionidae). Aquatic Biosystems, 8, 12. 22731520 10.1186/2046-9063-8-12 PMC3422191

[ece39717-bib-0013] Campbell, D. C. , Johnson, P. D. , Williams, J. D. , Rindsberg, A. K. , Serb, J. M. , Small, K. K. , & Lydeard, C. (2008). Identification of ‘extinct’ freshwater mussel species using DNA barcoding. Molecular Ecology Resources, 8, 711–724. 21585879 10.1111/j.1755-0998.2008.02108.x

[ece39717-bib-0014] Campbell, D. C. , & Lydeard, C. (2012a). Molecular systematics of *Fusconaia* (Bivalvia: Unionidae: Ambleminae). American Malacological Bulletin, 30, 1–17.

[ece39717-bib-0015] Campbell, D. C. , & Lydeard, C. (2012b). The genera of Pleurobemini (Bivalvia: Unionidae: Ambleminae). American Malacological Bulletin, 20, 19–38.

[ece39717-bib-0016] Campbell, D. C. , Serb, J. M. , Buhay, J. E. , Roe, K. J. , Minton, R. L. , & Lydeard, C. (2005). Phylogeny of north American amblemines (Bivalvia, Unionoida): Prodigious polyphyly proves pervasive across genera. Invertebrate Biology, 124, 131–164.

[ece39717-bib-0018] Culp, J. J. , Shepard, A. C. , & McGregor, M. A. (2009). Fish hosts and conglutinates of the pyramid pigtoe (*Pleurobema rubrum*). Southeastern Naturalist, 8, 19–22.

[ece39717-bib-0019] Cummings, K. , & Cordeiro, J. (2011). *Fusconaia flava*. The IUCN red list of threatened species 2011: E.T189275A8710330. 10.2305/IUCN.UK.2011-2.RLTS.T189275A8710330.en

[ece39717-bib-0020] Cummings, K. , & Cordeiro, J. (2012a). *Fusconaia subrotunda*. The IUCN red list of threatened species 2012: E.T8775A3146358. 10.2305/IUCN.UK.2012.RLTS.T8775A3146358.en

[ece39717-bib-0021] Cummings, K. , & Cordeiro, J. (2012b). *Pleurobema sintoxia*. The IUCN red list of threatened species 2012: E.T173061A1377100. 10.2305/IUCN.UK.2012.RLTS.T173061A1377100.en

[ece39717-bib-0023] Elderkin, C. L. (2009). Intragenomic variation in the rDNA internal transcribed spacer (ITS1) in the freshwater mussel *Cumberlandia monodonta* (say, 1828). Journal of Molluscan Studies, 75, 419–421.

[ece39717-bib-0024] Elkins, D. , Sweat, S. C. , Kuhajda, B. R. , George, A. L. , Hill, K. S. , & Wenger, S. J. (2019). Illuminating hotspots of imperiled aquatic biodiversity in the southeastern US. Global Ecology and Conservation, 19, e00654. 10.1016/j.gecco.2019.e00654

[ece39717-bib-0026] Folmer, O. , Black, M. , Hoeh, W. , Lutz, R. , & Vrijenhoek, R. (1994). DNA primers for amplification of mitochondrial cytochrome oxidase subunit I from diverse metazoan invertebrates. Molecular Marine Biology and Biotechnology, 3, 294–299. 7881515

[ece39717-bib-0027] Graf, D. L. , & Cummings, K. S. (2007). Review of the systematics and global diversity of freshwater mussel species (Bivalvia: Unionoida). Journal of Molluscan Studies, 73, 291–314.

[ece39717-bib-0028] Haag, W. R. (2012). North American freshwater mussels: Natural history, ecology, and conservation. Cambridge University Press.

[ece39717-bib-0029] Haag, W. R. , & Cicerello, R. R. (2016). A distributional atlas of the freshwater mussels of Kentucky (pp. 391–395). Kentucky State Nature Preserves Commission.

[ece39717-bib-0030] Haag, W. R. , & Warren, M. L. (2003). Host fishes and infection strategies of freshwater mussels in large Mobile Basin streams, USA. Journal of the North American Benthological Society, 22, 78–91.

[ece39717-bib-0031] Heard, W. H. , & Guckert, R. H. (1970). A re‐evaluation of the recent Unionacea (Pelecypoda) of North America. Malacologia, 10, 333–355.

[ece39717-bib-0032] Huson, D. H. , & Bryant, D. (2006). Application of phylogenetic networks in evolutionary studies. Molecular Biology and Evolution, 23, 254–267. 16221896 10.1093/molbev/msj030

[ece39717-bib-0033] Inoue, K. , Hayes, D. M. , Harris, J. L. , Johnson, N. A. , Morrison, C. L. , Eackles, M. S. , King, T. L. , Jones, J. W. , Hallerman, E. M. , Christian, A. D. , & Randklev, C. R. (2018). The Pleurobemini (Bivalvia: Unionida) revisited: Molecular species delineation using a mitochondrial DNA gene reveals multiple conspecifics and undescribed species. Invertebrate Systematics, 32, 689–702.

[ece39717-bib-0034] Inoue, K. , McQueen, A. L. , Harris, J. L. , & Berg, D. J. (2014). Molecular phylogenetics and morphological variation reveal recent speciation in freshwater mussels of genera *Acidens* and *Arkansia* (Bivalvia: Unionidae). Biological Journal of the Linnaean Society, 112, 535–545.

[ece39717-bib-0035] Jones, J. W. , Johnson, N. , Grobler, P. , Schilling, D. , Neves, R. J. , & Hallerman, E. M. (2015). Endangered rough pigtoe pearlymussel: Assessment of phylogenetic status and genetic differentiation of two disjunct populations. Journal of Fish and Wildlife Management, 6, 338–349.

[ece39717-bib-0036] Kimura, M. (1980). A simple method for estimating evolutionary rates of base substitutions through comparative studies of nucleotide sequences. Journal of Molecular Evolution, 16, 111–120. 7463489 10.1007/BF01731581

[ece39717-bib-0037] King, T. L. , Eackles, M. S. , Gjetvaj, B. , & Hoeh, W. R. (1999). Intraspecific phylogeography of *Lasmigona subviridis* (Bivalvia: Unionidae): Conservation implications of range discontinuity. Molecular Ecology, 8, S65–S78. 10703552 10.1046/j.1365-294x.1999.00784.x

[ece39717-bib-0039] Lopes‐Lima, M. , Burlakova, L. E. , Karatayev, A. Y. , Mehler, K. , Seddon, M. , & Sousa, R. (2018). Conservation of freshwater bivalves at the global scale: Diversity, threats and research needs. Hydrobiologia, 810, 1–14. 10.1007/s10750-017-3486-7

[ece39717-bib-0040] Löytynoja, A. , & Goldman, N. (2010). webPRANK: A phylogeny‐aware multiple sequence aligner with interactive alignment browser. BMC Bioinformatics, 11, 579. 21110866 10.1186/1471-2105-11-579 PMC3009689

[ece39717-bib-0041] Marshall, N. T. , Banta, J. A. , Williams, L. R. , Williams, M. G. , & Placyk, J. S. (2018). DNA barcoding permits identification of potential fish hosts of unionid freshwater mussels. American Malacological Bulletin, 36, 42–56.

[ece39717-bib-0042] Master, L. L. , Flack, S. R. , & Stein, B. A. (1998). Rivers of life: Critical watersheds for protecting freshwater biodiversity (p. 71). Nature Conservancy.

[ece39717-bib-0043] Miller, E. J. , Couch, K. J. , & Mason, J. (2008). A pocket guide to Kansas freshwater mussels (p. 72). Friends of the Great Plains Nature Center.

[ece39717-bib-0044] Morrison, C. , Johnson, N. , Jones, J. , Fitzgerald, D. , Aunins, A. , King, T. , & Hallerman, E. (2021). Genetic and morphological characterization of the clubshell species complex (*Pleurobema clava* and *P. oviforme*) to inform conservation planning. Ecology and Evolution, 12, 15325–15350. 10.1002/ece3.8219 PMC8571583 34765181

[ece39717-bib-0045] Nylander, J. A. A. (2008). MrModeltest 2.3. http://www.abc.se/~nylander/mrmodeltest2/mrmodeltest2.html

[ece39717-bib-0046] Olivera‐Hyde, M. O. (2021). Development of molecular and morphological resources for identification and monitoring of freshwater mussel species in the genera Fusconaia and Pleurobema in the Green River, Kentucky . [PhD Dissertation]. Virginia Polytechnic Institute and State University, Blacksburg, VA. https://vtechworks.lib.vt.edu/handle/10919/101843

[ece39717-bib-0100] Ortmann, A. E. (1920). Correlation of shape and station in fresh‐water mussels (Naiades). Proceedings of the American Philosophical Society, 59(4), 269–312.

[ece39717-bib-0050] Puillandre, N. , Lambert, A. , Brouillet, S. , & Achaz, G. (2012). ABGD, automatic barcode gap discovery for primary species delimitation. Molecular Ecology, 21, 1864–1877. 21883587 10.1111/j.1365-294X.2011.05239.x

[ece39717-bib-0051] Rambaut, A. (2014). FigTree v1. 4.2: Tree figure drawing tool. University of Edinburgh. http://tree.bio.ed.ac.uk/software/figtree/

[ece39717-bib-0052] Rambaut, A. , Suchard, M. A. , Xie, D. , & Drummond, A. J. (2009). MCMC trace analysis tool, version v1. 6.0. Institute of Evolutionary Biology, University of Edinburgh. http://tree.bio.ed.ac.uk/software/tracer/

[ece39717-bib-0053] Ronquist, F. , Teslenko, M. , Van Der Mark, P. , Ayres, D. L. , Darling, A. , Höhna, S. , Larget, B. , Liu, L. , Suchard, M. A. , & Huelsenbeck, J. P. (2012). MrBayes 3.2: Efficient Bayesian phylogenetic inference and model choice across a large model space. Systematic Biology, 61, 539–542. 22357727 10.1093/sysbio/sys029 PMC3329765

[ece39717-bib-0054] Rozas, J. , Librado, P. , Sanchez‐Delbarrio, J. C. , Messeguer, X. , & Rozas, R. (2009). DnaSP, version 5.10.00. Universita de Barcelona. http://www.ub.edu/dnasp/

[ece39717-bib-0055] Schilling, D. E. (2015). Assessment of morphological and molecular genetic variation of freshwater mussel species belonging to the genera Fusconaia, Pleurobema, and Pleuronaia in the upper Tennessee River basin . [M.S. Thesis, Virginia Tech]. pp. 1–6. https://vtechworks.lib.vt.edu/handle/10919/54030

[ece39717-bib-0056] Serb, J. M. , Buhay, J. E. , & Lydeard, C. (2003). Molecular systematics of the north American freshwater bivalve genus *Quadrula* (Unionidae: Ambleminae) based on mitochondrial *ND1* sequences. Molecular Phylogenetics and Evolution, 28, 1–11. 12801467 10.1016/s1055-7903(03)00026-5

[ece39717-bib-0057] Serb, J. M. , & Lydeard, C. (2003). Complete mtDNA sequence of the north American freshwater mussel, *Lampsilis ornata* (Unionidae): An examination of the evolution and phylogenetic utility of mitochondrial genome organization in Bivalvia (Mollusca). Molecular Biology and Evolution, 20, 1854–1866. 12949150 10.1093/molbev/msg218

[ece39717-bib-0058] Smith, C. H. , Johnson, N. A. , Pfeiffer, J. M. , & Gangloff, M. M. (2018). Molecular and morphological data to facilitate future research on freshwater mussels (Bivalvia: Unionidae: Anodontinae). Data in Brief, 17, 95–104. 29876378 10.1016/j.dib.2017.12.050 PMC5988452

[ece39717-bib-0060] Swofford, D. L. (1998). PAUP 4.0 beta version for windows: Phylogenetic analysis using parsimony. Sinauer Associates.

[ece39717-bib-0061] Tamura, K. , Stecher, G. , Peterson, D. , Filipski, A. , & Kumar, S. (2013). MEGA6: Molecular evolutionary genetics analysis version 6.0. Molecular Biology and Evolution, 30, 2725–2729. 24132122 10.1093/molbev/mst197 PMC3840312

[ece39717-bib-0062] Thompson, J. D. , Gibson, T. J. , & Higgins, D. G. (2003). Multiple sequence alignment using ClustalW and ClustalX. Current Protocols in Bioinformatics, 1, 2–3. 10.1002/0471250953.bi0203s00 18792934

[ece39717-bib-0063] U.S. Fish and Wildlife Service . (1976). Endangered status for 159 taxa of animals: 41 FR 24062–24067. http://ecos.fws.gov/speciesProfile/profile/speciesProfile.action?spcode=F00P#status

[ece39717-bib-0064] U.S. Fish and Wildlife Service . (1984). Rough pigtoe pearly mussel recovery plan. U.S. Fish and Wildlife Service, Atlanta, Georgia. Reference S1. http://ecos.fws.gov/speciesProfile/profile/speciesProfile.action?spcode=F00P

[ece39717-bib-0066] Walker, J. M. , Curole, J. P. , Wade, D. E. , Chapman, E. G. , Bogan, A. E. , Watters, G. T. , & Hoeh, W. R. (2006). Taxonomic distribution and phylogenetic utility of gender‐associated mitochondrial genomes in the Unionoida (Bivalvia). Malacologia, 48, 265–282.

[ece39717-bib-0067] Watters, G. T. (1994). Clubshell (Pleurobema clava) and northern riffleshell (Epioblasma torulosa rangiana) recovery plan (p. 67). Region five, U.S. Fish and Wildlife Service region 5 office. https://www.fws.gov/northeast/pafo/pdf/riffleshell_recovery_plan.pdf

