# Erratum

**DOI:** 10.1002/ece3.9822

**Published:** 2023-02-19

In the recent article by Olivera‐Hyde et al. (2023), the author contributions of the second author are incorrect. The correct details are shown below:

**Jess Jones:** Conceptualization (equal); funding acquisition (equal); investigation (equal); project administration (equal); resources (equal); supervision (equal); writing – review & editing (equal).

We apologize for these errors.
