# Supplementary material for: Phylogenetic assessment of endangered and look‐alike Pigtoe species in a freshwater mussel diversity hotspot
Source: Ecol Evol. 2023 Jan 24;13(1):e9717. doi: 10.1002/ece3.9717 (PMC9873586; doi:10.1002/ece3.9717)
Supplement: Supplementary file 1 — Appendix S1. [file ECE3-13-e9717-s001.docx]

**Supplemental Material**

**Supplemental Material (SM) 1 – Molecular methods**. For both pairs of *ND1* primers, PCR reactions were conducted in a volume of 22 µl which contained 0.45X GoTaq Flexi Buffer, 2.04 mM of MgCl_2_, 0.05 mM of each dNTP, 0.02 mg/ml of bovine serum albumin (BSA), 0.09 µM of each primer, 0.02 units/µl of GoTaq® DNA polymerase (Promega, Madison, WI), and 10-30 ng/µl of dsDNA template. The PCR protocol included an initial denaturation of 95^o^C for 5 min; followed by 30 cycles of 96^o^C for 20 sec, 53^o^C for 20 sec, and 72^o^C for 45 sec; a final extension at 72^o^C for 5 min; and a hold at 4^o^C.

PCR amplification for *COI* using primers LCO1490 (Folmer et al. 1994) and HCO700dy2 (Walker et al. 2006) was conducted in a volume of 22 µl which contained 0.45X GoTaq Flexi Buffer, 2.05 mM of MgCl_2_, 0.045 mM of each dNTPs, 0.02mg/ml of BSA, 0.09µM of each primer, 0.02 units/µl of GoTaq® DNA polymerase (Promega), and 10-30 ng/µl of dsDNA template. The PCR protocol included an initial denaturation of 94^o^C for 3 min; followed by 35 cycles of 94^o^C for 1 min, 57^o^C for 45 sec, and 72^o^C for 1 min; a final extension at 72^o^C for 5 min; and a hold at 4^o^C. Using primers COIF (Campbell et al. 2005) and HCO700dy2, the PCR reaction was conducted in a volume of 22 µl which contained 0.45X GoTaq Flexi Buffer, 2.27 mM of MgCl2, 0.072 mM of dNTP mix, 0.02 mg/ml of BSA, 0.018 µM of each primer, 0.02 units/µl of GoTaq® DNA polymerase (Promega), and 10-30 ng/µl of dsDNA template. The PCR protocol included an initial denaturation of 94^o^C for 3 min; followed by 35 cycles of 94^o^C for 1 min, 55^o^C for 45 sec, and 72oC for 1 min; a final extension at 72^o^C for 5 min; and a hold at 4^o^C.

For *ITS-1*, PCR reactions were conducted in a final volume of 22 µl which contained 0.45X GoTaq Flexi Buffer, 2.05 mM of MgCl2, 0.05 mM of each dNTPs, 0.02 mg/ml of BSA, 0.09 µM of each primer, 0.02 units/µl of GoTaq® DNA polymerase (Promega), and 10-30 ng/µl of dsDNA. The PCR protocol included an initial denaturation of 94^o^C for 5 min; followed by 35 cycles of 94oC for 40 sec, 64^o^C for 40 sec, and 72^o^C for 1 min; a final extension at 72^o^C for 5 min; and a hold at 4^o^C.

**REFERNECES**

Campbell, D. C., Serb, J. M., Buhay, J.E.., Roe, K.J., Minton, R.L., & Lydeard, C. (2005). Phylogeny of North American amblemines (Bivalvia, Unionoida): prodigious polyphyly proves pervasive across genera. *Invertebrate Biology,* *124,*131-164.

Folmer, O., Black, M., Hoeh, W., Lutz, R., & Vrijenhoek, R. (1994). DNA primers for amplification of mitochondrial cytochrome oxidase subunit I from diverse metazoan invertebrates. *Molecular Marine Biology and Biotechnology,* *3*, 294-299.

Walker, J. M., Curole, J.P., Wade, D.E. Chapman, E.G., Bogan, A.E., Watters, G.T., & Hoeh, W.R. (2006). Taxonomic distribution and phylogenetic utility of gender-associated mitochondrial genomes in the Unionoida (Bivalvia). *Malacologia,* *48*, 265-282.

**SM 2 - GenBank accession numbers and sampling locations for DNA sequences added to the phylogenetic analysis.**

SM 2 Table S1 The mitochondrial DNA *COI* gene sequences from Inoue et al. (2018) that were added to the phylogenetic tree and split network analyses.

The sequences include the species (Fask = *Fusconaia askewi*, Flan = *Fusconaia lanensis*, Fcer = *Fusconaia cerina*, Fmas = *Fusconaia masoni*, Fsub = *Fusconaia subrotunda*, Fchu = *Fusconaia chunii*, Pcor = *Pleurobema cordatum*, Pple = *Pleurobema plenum*, Prid = *Pleurobema riddellii*, Prub = *Pleurobema rubrum*, and Psin = *Pleurobema sintoxia*) followed their respective GenBank accession number and their state location. After reference sequences were trimmed to match the length of my Green River sequences, they grouped together. These are reported as similar sequences.

| Species | Sequences added in the tree | Similar sequences |
| --- | --- | --- |
| *Fusconaia askewi* | Fask_MF961812_TX |  |
| *Fusconaia askewi* | Fask_MF961814_TX | Flan_MF961935_TX |
| *Fusconaia askewi* | Fask_MF961815_TX | Flan_MF961932_TX |
| *Fusconaia askewi* | Fask_MF961817_TX |  |
| *Fusconaia askewi* | Fask_MF961818_TX | Fask_MF961826_TX, Flan_MF961931_TX |
| *Fusconaia askewi* | Fask_MF961819_TX | Fask_MF961821_TX, Fask_MF961831_TX, Fask_MF961838_TX |
| *Fusconaia askewi* | Fask_MF961825_TX |  |
| *Fusconaia askewi* | Fask_MF961828_TX | Fask_MF961830_TX |
| *Fusconaia askewi* | Fask_MF961832_TX |  |
| *Fusconaia askewi* | Fask_MF961836_TX |  |
| *Fusconaia askewi* | Fask_MF961839_TX | Fask_MF961840_TX |
| *Fusconaia askewi* *Fusconaia lanensis* | Fask_MF961813_TX | Fask_MF961816_TX, Fask_MF961820_TX, Fask_MF961823_TX, Fask_MF961824_TX, Fask_MF961827_TX, Fask_MF961829_TX, Fask_MF961834_TX, Fask_MF961835_TX, Fask_MF961837_TX, Flan_MF961933_TX, Flan_MF961934_TX, Flan_MF961936_TX, Flan_MF961937_TX, Flan_MF961938_TX, |
| *Fusconaia cerina* | Fcer_MF961846_LA |  |
| *Fusconaia cerina* | Fcer_MF961848_LA |  |
| *Fusconaia cerina* | Fcer_MF961849_MS |  |
| *Fusconaia cerina* | Fcer_MF961850_MS | Ffla_MF961896_LA, Ffla_MF961900_AL |
| *Fusconaia cerina* | Fcer_MF961851_AL | Fcer_MF961852_AL |
| *Fusconaia cerina* *Fusconaia flava* | Fcer_MF961843_LA | Ffla_MF961879_AR, Ffla_MF961883_AR, Ffla_MF961885_AR, Ffla_MF961886_AR, Ffla_MF961887_AR, Ffla_MF961888_AR, Ffla_MF961889_AR, Ffla_MF961890_AR, Ffla_MF961895_OK, Ffla_MF961907_OH, Ffla_MF961908_OH, Ffla_MF961918_AR, Ffla_MF961919_AR, Ffla_MF961923_KY_IN, Ffla_MF961925_AR, Ffla_MF961926_AR |
| *Fusconaia cerina* *Fusconaia flava* | Fcer_MF961844_LA | Fcer_MF961845_LA, Fcer_MF961847_LA, Ffla_MF961913_AR |
| *Fusconaia flava* | Ffla_MF961867_AR |  |
| *Fusconaia flava* | Ffla_MF961869_AR |  |
| *Fusconaia flava* | Ffla_MF961870_AR |  |
| *Fusconaia flava* | Ffla_MF961871_AR | Ffla_MF961872_AR, Ffla_MF961874_AR |
| *Fusconaia flava* | Ffla_MF961873_AR |  |
| *Fusconaia flava* | Ffla_MF961875_AR |  |
| *Fusconaia flava* | Ffla_MF961876_KY | Ffla_MF961877_KY, Ffla_MF961878_KY, Ffla_MF961917_AR |
| *Fusconaia flava* | Ffla_MF961880_MN |  |
| *Fusconaia flava* | Ffla_MF961881_AR | Ffla_MF961882_AR, Ffla_MF961884_AR, Ffla_MF961927_AR |
| *Fusconaia flava* | Ffla_MF961891_TX | Ffla_MF961893_TX, Ffla_MF961904_AR |
| *Fusconaia flava* | Ffla_MF961892_TX |  |
| *Fusconaia flava* | Ffla_MF961894_AR |  |
| *Fusconaia flava* | Ffla_MF961897_LA |  |
| *Fusconaia flava* | Ffla_MF961898_AR |  |
| *Fusconaia flava* | Ffla_MF961899_LA | Ffla_MF961910_AR |
| *Fusconaia flava* | Ffla_MF961901_LA | Ffla_MF961902_LA |
| *Fusconaia flava* | Ffla_MF961903_AR | Ffla_MF961920_AR |
| *Fusconaia flava* | Ffla_MF961905_AR |  |
| *Fusconaia flava* | Ffla_MF961906_AR |  |
| *Fusconaia flava* | Ffla_MF961911_AR |  |
| *Fusconaia flava* | Ffla_MF961912_AR |  |
| *Fusconaia flava* | Ffla_MF961914_AR |  |
| *Fusconaia flava* | Ffla_MF961915_AR |  |
| *Fusconaia flava* | Ffla_MF961916_AR |  |
| *Fusconaia flava* | Ffla_MF961922_AR |  |
| *Fusconaia flava* | Ffla_MF961924_LA |  |
| *Fusconaia flava* | Ffla_MF961928_AR |  |
| *Fusconaia flava* | Ffla_MF961929_AR |  |
| *Fusconaia flava* | Ffla_MF961930_AR |  |
| *Fusconaia masoni* | Fmas_MF961939_VA |  |
| *Fusconaia masoni* | Fmas_MF961940_VA |  |
| *Fusconaia masoni* | Fmas_MF961941_NC | Fmas_MF961942_NC |
| *Fusconaia subrotunda* | Fsub_MF961948_KY |  |
| *Fusconaia subrotunda* | Fsub_MF961949_KY | Fsub_MF961950_KY |
| *Fusconaia subrotunda* | Fsub_MF961951_NC |  |
| *Fusconaia subrotunda* | Fsub_MF961952_NC |  |
| *Fusconaia subrotunda* | Fsub_MF961953_NC |  |
| *Fusconaia subrotunda* | Fsub_MF961954_NC |  |
|  |  |  |

SM 2 Table S1. Continued.

| Species | Sequences added in the tree | Similar sequences |
| --- | --- | --- |
| *Fusconia chunii* | Fchu_MF961853_TX | Fchu_MF961854_TX, Fchu_MF961855_TX, Fchu_MF961856_TX |
| *Pleurobema cordatum* | Pcor_MF961959_KY |  |
| *Pleurobema cordatum* | Pcor_MF961960_KY | Pcor_MF961963_KY |
| *Pleurobema cordatum* | Pcor_MF961961_KY | Pcor_MF961965_OH, Pcor_MF961968_OH |
| *Pleurobema cordatum* | Pcor_MF961962_KY |  |
| *Pleurobema cordatum* | Pcor_MF961964_OH |  |
| *Pleurobema cordatum* | Pcor_MF961966_AR |  |
| *Pleurobema cordatum* | Pcor_MF961967_OH |  |
| *Pleurobema cordatum* | Pcor_MF961969_AL |  |
| *Pleurobema dolabelloides* | Pdol_MF962140 |  |
| *Pleurobema plenum* | Pple_MF961970_TN | Pple_MF961972_TN |
| *Pleurobema plenum* | Pple_MF961971_TN |  |
| *Pleurobema plenum* | Pple_MF961973_TN |  |
| *Pleurobema riddellii* | Prid_MF961974_AR | Prid_MF961976_AR, Prid_MF961978_AR, Prid_MF961979_AR, Prid_MF961981_AR, Prid_MF961982_AR, Prid_MF961983_AR, Prid_MF961984_AR, Prid_MF961986_AR, Prid_MF961988_AR |
| *Pleurobema riddellii* | Prid_MF961975_AR | Prid_MF961977_AR |
| *Pleurobema riddellii* | Prid_MF961980_AR |  |
| *Pleurobema riddellii* | Prid_MF961985_AR |  |
| *Pleurobema riddellii* | Prid_MF961987_AR |  |
| *Pleurobema riddellii* | Prid_MF961989_AR |  |
| *Pleurobema riddellii* | Prid_MF961990_AR | Prid_MF961991_AR |
| *Pleurobema riddellii* | Prid_MF961993_AR |  |
| *Pleurobema riddellii* | Prid_MF961994_TX | Prid_MF961995_TX, Prid_MF961996_TX, Prid_MF961997_TX, Prid_MF962002_TX, Prid_MF962003_TX, Prid_MF962004_TX |
| *Pleurobema riddellii* | Prid_MF961998_TX |  |
| *Pleurobema riddellii* | Prid_MF961999_TX | Prid_MF962000_TX, Prid_MF962001_TX |
| *Pleurobema rubrum* | Prub_MF962005_TN |  |
| *Pleurobema rubrum* | Prub_MF962006_TN |  |
| *Pleurobema rubrum* | Prub_MF962007_KY |  |
| *Pleurobema rubrum* | Prub_MF962010_AR |  |
| *Pleurobema rubrum* | Prub_MF962012_AR |  |
| *Pleurobema rubrum* | Prub_MF962013_AR |  |
| *Pleurobema rubrum* | Prub_MF962014_AR |  |
| *Pleurobema rubrum* | Prub_MF962015_AR |  |
| *Pleurobema rubrum* | Prub_MF962018_AR |  |

SM 2. Table S1. Continued.

| Species | Sequences added in the tree | Similar sequences |
| --- | --- | --- |
| *Pleurobema rubrum* | Prub_MF962021_AR |  |
| *Pleurobema rubrum* | Prub_MF962024_AR |  |
| *Pleurobema rubrum* | Prub_MF962026_AR |  |
| *Pleurobema rubrum* | Prub_MF962029_AR |  |
| *Pleurobema rubrum* | Prub_MF962030_AR |  |
| *Pleurobema rubrum* | Prub_MF962033_TN |  |
| *Pleurobema rubrum* *Pleurobema sintoxia* | Prub_MF962008_AR | Prub_MF962016_AR, Prub_MF962020_AR, Prub_MF962025_AR, Prub_MF962027_AR, Psin_MF962035_AR, Psin_MF962064_AR, Psin_MF962065_AR, Psin_MF962080_AR, Psin_MF962081_AR, Psin_MF962087_AR |
| *Pleurobema rubrum* *Pleurobema sintoxia* | Prub_MF962009_AR | Prub_MF962011_AR, Prub_MF962017_AR, Prub_MF962022_AR, Prub_MF962028_AR, Psin_MF962038_TN, Psin_MF962039_TN, Psin_MF962043_MN, Psin_MF962050_KY, Psin_MF962053_KY, Psin_MF962054_PA, Psin_MF962055_PA, Psin_MF962069_AR, Psin_MF962076_KY, Psin_MF962079_AR, Psin_MF962084_AR, Psin_MF962085_AR, Psin_MF962092_AR, Psin_MF962097_AR |
| *Pleurobema rubrum* *Pleurobema sintoxia* | Prub_MF962019_AR | Psin_MF962090_AR, Psin_MF962091_AR |
| *Pleurobema rubrum* *Pleurobema sintoxia* | Prub_MF962031_TN | Psin_MF962045_KY |
| *Pleurobema rubrum* *Pleurobema sintoxia* | Prub_MF962032_TN | Psin_MF962056_PA, Psin_MF962061_PA, Psin_MF962073_TN, Psin_MF962074_TN, Psin_MF962075_TN |
| *Pleurobema rubrum* *Pleurobema sintoxia* | Prub_MF962034_AR | Psin_MF962094_AR, Psin_MF962095_AR, Psin_MF962096_AR, Psin_MF962098_AR, Psin_MF962099_AR, Psin_MF962101_AR, Psin_MF962102_AR, Psin_MF962103_AR |
| *Pleurobema sintoxia* | Psin_MF962036_AR |  |
| *Pleurobema sintoxia* | Psin_MF962037_TN | Psin_MF962072_TN |
| *Pleurobema sintoxia* | Psin_MF962040_PA |  |
| *Pleurobema sintoxia* | Psin_MF962041_WI |  |
| *Pleurobema sintoxia* | Psin_MF962042_WI |  |
| *Pleurobema sintoxia* | Psin_MF962046_KY | Psin_MF962060_PA |
| *Pleurobema sintoxia* | Psin_MF962047_KY | Psin_MF962049_KY |
| *Pleurobema sintoxia* | Psin_MF962048_KY |  |
| *Pleurobema sintoxia* | Psin_MF962051_KY | Psin_MF962052_KY |
| *Pleurobema sintoxia* | Psin_MF962057_PA | Psin_MF962058_PA, Psin_MF962059_PA |
| *Pleurobema sintoxia* | Psin_MF962062_AR | Psin_MF962104_AR |
| *Pleurobema sintoxia* | Psin_MF962063_AR |  |
| *Pleurobema sintoxia* | Psin_MF962066_AR |  |

SM 2 Table S1. Continued.

| Species | Sequences added in the tree | Similar sequences |
| --- | --- | --- |
| *Pleurobema sintoxia* | Psin_MF962067_AR |  |
| *Pleurobema sintoxia* | Psin_MF962068_AR |  |
| *Pleurobema sintoxia* | Psin_MF962070_AR |  |
| *Pleurobema sintoxia* | Psin_MF962071_AR |  |
| *Pleurobema sintoxia* | Psin_MF962077_KY | Psin_MF962078_KY |
| *Pleurobema sintoxia* | Psin_MF962082_AR |  |
| *Pleurobema sintoxia* | Psin_MF962083_AR |  |
| *Pleurobema sintoxia* | Psin_MF962086_AR |  |
| *Pleurobema sintoxia* | Psin_MF962088_AR |  |
| *Pleurobema sintoxia* | Psin_MF962100_AR |  |

SM 2 Table S2. Sequences from other studies utilizing the mitochondrial DNA *ND1* gene that were added to the phylogenetic tree and split network analyses.

The sequences include the species (Fask = *Fusconaia askewi*, Flan = *Fusconaia lanensis*, Fmas = *Fusconaia masoni*, Fsub = *Fusconaia subrotunda*, Pcor = *Pleurobema cordatum*, Pple = *Pleurobema plenum*, Prub = *Pleurobema rubrum*, Psin = *Pleurobema sintoxia*, Pdol = *Pleurobema dolabelloides*) followed their respective GenBank accession number or haplotype name for Jones et al. 2015 sequences, and their sampling location.

| Species | Name in the tree | Waterbody | Reference |
| --- | --- | --- | --- |
| *Fusconaia askewi* | Fask_JN180976_TX | Sabine drainage, Texas | Burlakova et al. 2012 |
| *Fusconaia askewi* | Fask_JN180977_TX | Sabine drainage, Texas | Burlakova et al. 2012 |
| *Fusconaia askewi* | Fask_KY442832_TX | Texas | Bertram et al. 2015 |
| *Fusconaia askewi* | Fask_KY442833_TX | Texas | Bertram et al. 2015 |
| *Fusconaia askewi* | Fask_MG020448_TX | Texas | Marshall el al. 2018 |
| *Fusconaia lanensis* | Flan_JN180980_TX | Texas | Burlakova et al. 2012 |
| *Fusconaia lanensis* | Flan_JN180982_TX | Texas | Burlakova et al. 2012 |
| *Fusconaia masoni* | Fmas_KT187973_VA | Craig Creek, Virginia | Schilling 2015 |
| *Fusconaia masoni* | Fmas_KT187974_VA | Craig Creek, Virginia | Schilling 2015 |
| *Pleurobema rubrum* | Prub_KT188097_DR | Duck River, Tennessee | Schilling 2015 |
| *Pleurobema rubrum* | Prub_KT188096_DR | Duck River, Tennessee | Schilling 2015 |
| *Pleurobema rubrum* | Prub_KT188095_CL | Clinch Drainage, Tennessee | Schilling 2015 |
| *Fusconaia subrotunda* | Fsub_KT187998_ND | Nolichucky Drainage, Tennessee | Schilling 2015 |
| *Fusconaia subrotunda* | Fsub_KT187997_CL | Clinch Drainage, Tennessee | Schilling 2015 |
| *Fusconaia subrotunda* | Fsub_KT187996_CL | Clinch Drainage, Tennessee | Schilling 2015 |
| *Fusconaia subrotunda* | Fsub_KT187995_CL | Clinch Drainage, Tennessee | Schilling 2015 |
| *Fusconaia subrotunda* | Fsub_KT187994_CL | Clinch Drainage, Tennessee | Schilling 2015 |
| *Fusconaia subrotunda* | Fsub_KT187993_CL | Clinch Drainage, Tennessee | Schilling 2015 |
| *Fusconaia subrotunda* | Fsub_KT187992_CL | Clinch Drainage, Tennessee | Schilling 2015 |
| *Fusconaia subrotunda* | Fsub_KT187991_CL | Clinch Drainage, Tennessee | Schilling 2015 |
| *Fusconaia subrotunda* | Fsub_KT187990_CL | Clinch Drainage, Tennessee | Schilling 2015 |
| *Fusconaia subrotunda* | Fsub_KT187989_CL | Clinch Drainage, Tennessee | Schilling 2015 |
| *Fusconaia subrotunda* | Fsub_KT187988_PO | Powell Drainage, Tennessee | Schilling 2015 |
| *Fusconaia subrotunda* | Fsub_KT187987_CL | Powell Drainage, Tennessee | Schilling 2015 |
| *Fusconaia subrotunda* | Fsub_KT187986_PO | Powell Drainage, Tennessee | Schilling 2015 |
| *Fusconaia subrotunda* | Fsub_KT187985_PO | Powell Drainage, Tennessee | Schilling 2015 |
| *Fusconaia subrotunda* | Fsub_KT187984_PO | Powell Drainage, Tennessee | Schilling 2015 |
| *Fusconaia subrotunda* | Fsub_KT187983_PO | Powell Drainage, Tennessee | Schilling 2015 |
| *Fusconaia subrotunda* | Fsub_KT187982_CL | Clinch Drainage, Tennessee | Schilling 2015 |
| *Fusconaia subrotunda* | Fsub_KT187981_CL | Clinch Drainage, Tennessee | Schilling 2015 |
| *Fusconaia subrotunda* | Fsub_KT187980_CL | Clinch Drainage, Tennessee | Schilling 2015 |
| *Fusconaia subrotunda* | Fsub_KT187979_CL | Clinch Drainage, Tennessee | Schilling 2015 |
| *Fusconaia subrotunda* | Fsub_KT187978_CL | Clinch Drainage, Tennessee | Schilling 2015 |
| *Fusconaia subrotunda* | Fsub_KT187977_CL | Clinch Drainage, Tennessee | Schilling 2015 |
| *Fusconaia subrotunda* | Fsub_KT187976_CL | Clinch Drainage, Tennessee | Schilling 2015 |

SM 2 Table S2. Continued.

| Species | Name in the tree | Waterbody | Reference |
| --- | --- | --- | --- |
| *Fusconaia subrotunda* | Fsub_KT187975_CL | Clinch Drainage | Schilling 2015 |
| *Pleurobema cordatum* | Pcor_PcGreen01_GR | Green River, Kentucky | Jones et al. 2015 |
| *Pleurobema cordatum* | Pcor_PcGreen02_GR | Green River, Kentucky | Jones et al. 2015 |
| *Pleurobema cordatum* | Pcor_PcGreen03_GR | Green River, Kentucky | Jones et al. 2015 |
| *Pleurobema cordatum* | Pcor_PcGreen04_GR | Green River, Kentucky | Jones et al. 2015 |
| *Pleurobema cordatum* | Pcor_PcGreen05_GR | Green River, Kentucky | Jones et al. 2015 |
| *Pleurobema cordatum* | Pcor_PcGreen06_GR | Green River, Kentucky | Jones et al. 2015 |
| *Pleurobema cordatum* | Pcor_PcGreen07_GR | Green River, Kentucky | Jones et al. 2015 |
| *Pleurobema cordatum* | Pcor_PcGreen08_GR | Green River, Kentucky | Jones et al. 2015 |
| *Pleurobema cordatum* | Pcor_PcGreen09_GR | Green River, Kentucky | Jones et al. 2015 |
| *Pleurobema cordatum* | Pcor_PcGreen10_GR | Green River, Kentucky | Jones et al. 2015 |
| *Pleurobema cordatum* | Pcor_PcGreen11_GR | Green River, Kentucky | Jones et al. 2015 |
| *Pleurobema cordatum* | Pcor_PcGreen12_GR | Green River, Kentucky | Jones et al. 2015 |
| *Pleurobema cordatum* | Pcor_PcGreen13_GR | Green River, Kentucky | Jones et al. 2015 |
| *Pleurobema cordatum* | Pcor_PcTenn14_TN | Tennessee River Basin | Jones et al. 2015 |
| *Pleurobema cordatum* | Pcor_PcTenn15_TN | Tennessee River Basin | Jones et al. 2015 |
| *Pleurobema plenum* | Pple_PpClinch01_CL | Clinch River, Tennessee | Jones et al. 2015 |
| *Pleurobema plenum* | Pple_PpClinch02_CL | Clinch River, Tennessee | Jones et al. 2015 |
| *Pleurobema plenum* | Pple_PpClinch03_CL | Clinch River, Tennessee | Jones et al. 2015 |
| *Pleurobema plenum* | Pple_PpClinch04_CL | Clinch River, Tennessee | Jones et al. 2015 |
| *Pleurobema plenum* | Pple_PpClinch05_CL | Clinch River, Tennessee | Jones et al. 2015 |
| *Pleurobema plenum* | Pple_PpClinch06_CL | Clinch River, Tennessee | Jones et al. 2015 |
| *Pleurobema plenum* | Pple_PpClinch07_CL | Clinch River, Tennessee | Jones et al. 2015 |
| *Pleurobema plenum* | Pple_PpClinch08_CL | Clinch River, Tennessee | Jones et al. 2015 |
| *Pleurobema plenum* | Pple_PpClinch09_CL | Clinch River, Tennessee | Jones et al. 2015 |
| *Pleurobema plenum* | Pple_PpClinch10_CL | Clinch River, Tennessee | Jones et al. 2015 |
| *Pleurobema plenum* | Pple_PpClinch11_CL | Clinch River, Tennessee | Jones et al. 2015 |
| *Pleurobema plenum* | Pple_PpGreen12_GR | Green River, Kentucky | Jones et al. 2015 |
| *Pleurobema plenum* | Pple_PpGreen13_GR | Green River, Kentucky | Jones et al. 2015 |
| *Pleurobema plenum* | Pple_PpGreen14_GR | Green River, Kentucky | Jones et al. 2015 |
| *Pleurobema plenum* | Pple_PpGreen15_GR | Green River, Kentucky | Jones et al. 2015 |
| *Pleurobema plenum* | Pple_PpGreen16_GR | Green River, Kentucky | Jones et al. 2015 |
| *Pleurobema rubrum* | Prub_PrClinch01_CL | Clinch River, Tennessee | Jones et al. 2015 |
| *Pleurobema rubrum* | Prub_PrClinch02_CL | Clinch River, Tennessee | Jones et al. 2015 |
| *Pleurobema rubrum* | Prub_PrClinch03_CL | Clinch River, Tennessee | Jones et al. 2015 |
| *Pleurobema sintoxia* | Psin_PsGreen01_GR | Green River, Kentucky | Jones et al. 2015 |
| *Pleurobema sintoxia* | Psin_PsGreen02_GR | Green River, Kentucky | Jones et al. 2015 |
| *Pleurobema dolabelloides* | Pdol_KT188034 |  | Schilling 2015 |

**Supplemental Material 3. Phylogenetic trees reconstructed for mitochondrial markers *COI* and *ND1***


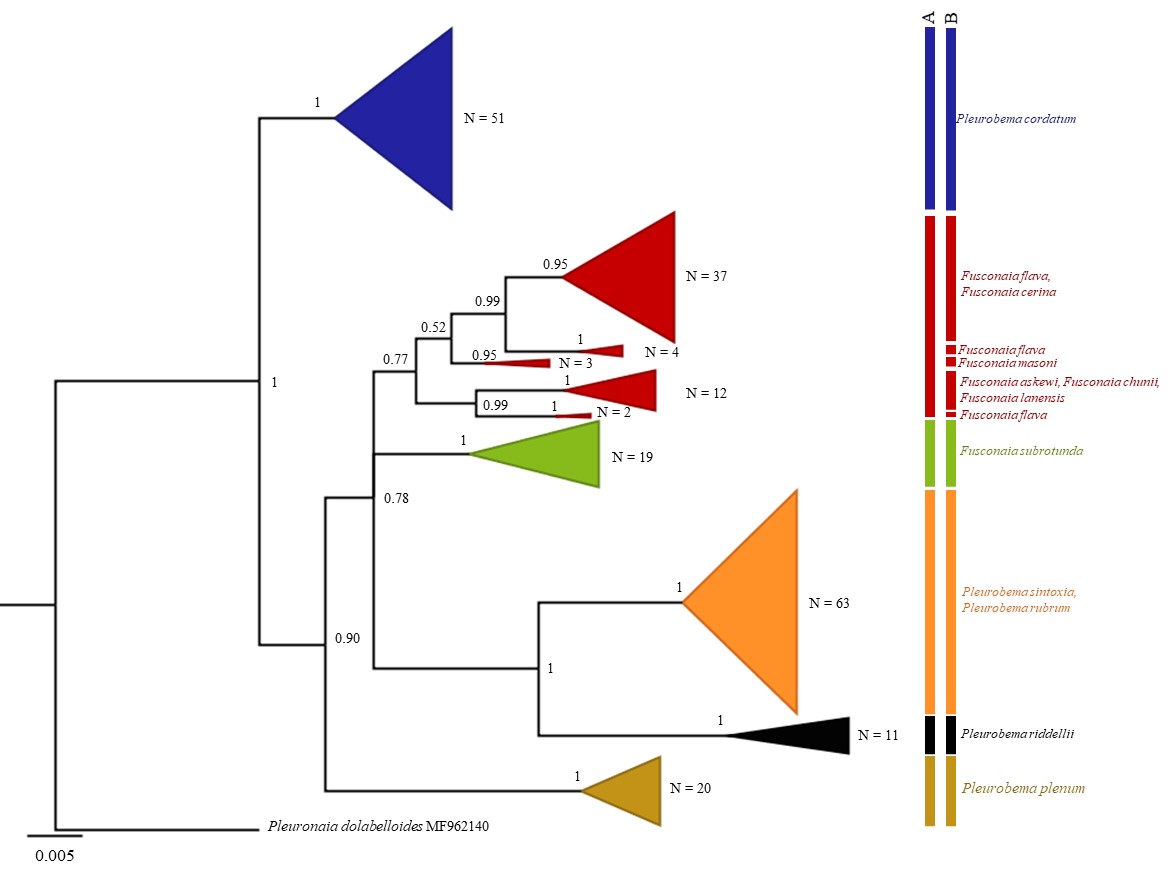


SM 3 Figure S1. Phylogenetic tree constructed using mitochondrial DNA *COI* sequences and Bayesian consensus trees in MrBayes.

The most appropriate model of nucleotide substitution using the Akaike Information Criterion (AIC) was the General Time Reversible (GTR+G+I) model that followed a gamma distribution with a proportion of invariable sites. The analysis was run with 11 million generations and trees were sampled every 250 generations, which generated a total of 66,002 trees. The final standard deviation of split frequencies was 0.009889 with a –ln likelihood of -3386.51. Posterior probabilities are indicated to the left of the respective nodes. The outgroup was *Pleuronaia dolabelloides* (MF962140). Species differentiation was assessed using the Automatic Barcode Gap Discovery (ABGD). To assign mussel specimens into the different hypothetical species, the Kimura (1980) two-parameter (K2P) distance model was used, where the minimum intraspecific genetic distance (*P_min_*) was set at 0.001 and the maximum intraspecific genetic distance (*P_max_*) was set at 0.1. Two partitions for species delimitation, partition (A), has a prior maximal distance of P = 4.64 e^-3^ while partition B with a maximal distance P = 1.00 e^-3^. Mussel specimens were collected in 2015 and 2017 from Pool 4 (GPS coordinates = 37.18286, -86.6296; river mile = 149) and Mammoth Cave National Park (GPS coordinates 37.17819, -86.1154; river mile = 197) in the Green River, Kentucky. Additional mussel specimens were collected from the Clinch River, Hancock County, TN, and the Tennessee River downstream of Pickwick dam, Hardin County, TN. Additional sequences for *Fusconaia askewi*, *F. chunii*, *F. cerina*, *F. cerina*, *F. flava*, *F. lanensis*, *F. masoni*, *F. subrotunda*, *Pleurobema cordatum*, *P. plenum*, *P. riddellii*, *P. rubrum*, and *P. sintoxia* were obtained from Inoue et al. (2018), With their respective GenBank accession numbers available in SM Table 1.


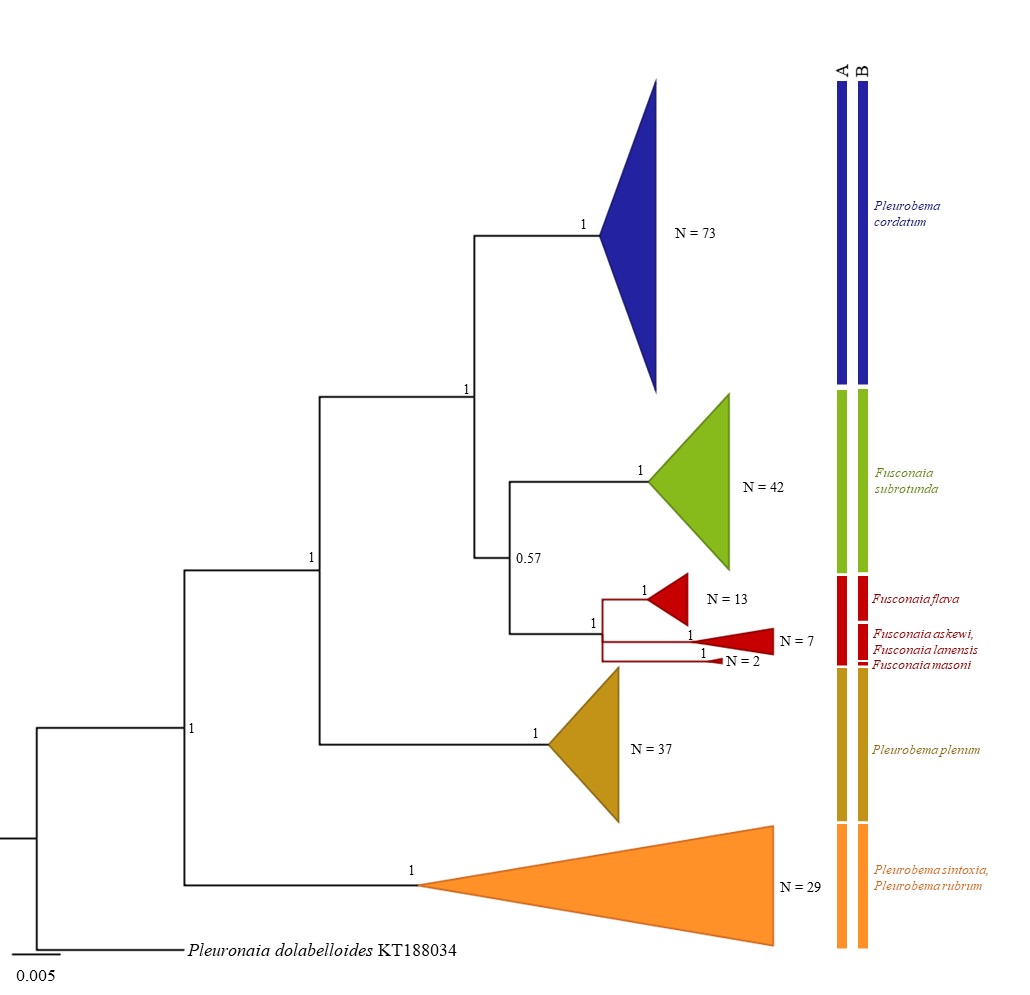


SM 3 Figure S2. Phylogenetic tree constructed using mitochondrial DNA *ND1* sequences and Bayesian consensus trees in MrBayes.

The most appropriate model of nucleotide substitution using the Akaike Information Criterion (AIC) was the General Time Reversible (GTR+G+I) model that followed a gamma distribution with a proportion of invariable sites. The analysis was run with 11 million generations and trees were sampled every 1000 generations, which generated a total of 16502 trees. The final standard deviation of split frequencies was 0.007619 with a –ln likelihood of -4394.69. Posterior probabilities are indicated next to the respective nodes. The outgroup was *Pleuronaia dolabelloides* (KT188034). Species differentiation was assessed using the Automatic Barcode Gap Discovery (ABGD). Recurse partition was labeled as ABGD*. In this software, to assign mussel specimens into the different hypothetical species, the Kimura (1980) two-parameter (K2P) distance model was used, where the minimum intraspecific genetic distance (*P_min_*) was set at 0.001 and the maximum intraspecific genetic distance (*P_max_*) was set at 0.1. Two partitions for species delimitation, partition (A), has a prior maximal distance of *P* = 4.64 e^-3^ and partition B is its recursive partition with the same prior maximal distance. Mussel specimens were collected in 2015 and 2017 from Pool 4 (GPS coordinates = 37.18286, -86.6296; river mile = 149) and Mammoth Cave National Park (GPS coordinates 37.17819, -86.1154; river mile = 197) in the Green River, Kentucky. Additional mussel specimens were collected from the Clinch River, Hancock County, TN, and Tennessee River downstream of Pickwick dam, Hardin County, TN. Additional reference sequences for *Fusconaia askewi*, *F. lanensis*, *F. masoni*, *F. subrotunda*, *Pleurobema cordatum*, *P. plenum*, and *P. sintoxia* were obtained from Bertram et al. 2015, Burlakova et al. 2012, Jones et al. 2015, Marshall et al. 2018, and Schilling 2015 with their respective accession numbers available in SM Table 2.
